# Delivering Cas9/sgRNA ribonucleoprotein (RNP) by lentiviral capsid-based bionanoparticles for efficient ‘hit-and-run’ genome editing

**DOI:** 10.1093/nar/gkz605

**Published:** 2019-07-12

**Authors:** Pin Lyu, Parisa Javidi-Parsijani, Anthony Atala, Baisong Lu

**Affiliations:** 1 College of Life Sciences, Anhui Normal University, Wuhu, Anhui, 241000, China; 2 Wake Forest Institute for Regenerative Medicine, Wake Forest University Health Sciences, Winston-Salem, NC, 27101, USA

## Abstract

Transient expression of the CRISPR/Cas9 machinery will not only reduce risks of mutagenesis from off-target activities, but also decrease possible immune response to Cas9 protein. Building on our recent developing of a system able to package up to 100 copies of *Cas9* mRNA in each lentivirus-like particle (LVLP) via the specific interaction between aptamer and aptamer-binding proteins (ABP), here we develop a lentiviral capsid-based bionanoparticle system, which allows efficient packaging of Cas9/sgRNA ribonucleoprotein (RNP). We show that replacing the Tetraloop of sgRNA scaffold with a *com* aptamer preserves the functions of the guide RNA, and the *com*-modified sgRNA can package Cas9/sgRNA RNP into lentivirus-like particles via the specific interactions between ABP and aptamer, and sgRNA and Cas9 protein. These RNP bionanoparticles generated Indels on different targets in different cells with efficiencies similar to or better than our recently described *Cas9* mRNA LVLPs. The new system showed fast action and reduced off-target rates, and makes it more convenient and efficient in delivering Cas9 RNPs for transient Cas9 expression and efficient genome editing.

## INTRODUCTION

Transient expression of the CRISPR/Cas9 machinery is important for several reasons, including the possibility of off-targets (1,2), the positive relationship between the off-target rates and the durations of the CRISPR/Cas9 action (3), and the high prevalence of Cas9 immune responses in the adult human population (4,5). Transient expression of the CRISPR/Cas9 machinery will not only reduce off-targets, but also minimize possible immune response from the host, which leads to improved safety and efficiency of gene editing. Therefore, strategies for transient delivery of the CRISPR/Cas9 components have been explored, including delivering Cas9/single guide RNA (sgRNA) ribonucleoprotein (RNP) by electroporation (6), conjugating Cas9 protein to cell-penetrating peptides (7) and delivering Cas9 RNP by cationic lipid (8) or gold nanoparticles (9).

Lentiviral vector is a widely used gene delivery vehicle in research laboratories and many *ex vivo* gene therapy clinical trials (https://clinicaltrials.gov). It is also widely used for delivering the CRISPR/Cas9 machinery for efficient genome editing (3,10). An obvious disadvantage of the lentiviral vectors for gene editing is that they mediate long-term expression of the CRISPR/Cas9 machinery, which could be problematic in clinical applications. Delivering Cas9 protein with lentivirus-like particles enabled the transient expression but only showed moderate efficiency of particle production and gene editing (11). Utilizing the specific interactions between aptamer and aptamer-binding protein (ABP), we recently developed a lentivirus-like particle (LVLP) system for *Cas9* mRNA delivery (12). Compared with similar efforts using lentivirus-like particles for mRNA delivery (13,14), our LVLP system showed greatly increased particle yield, cargo RNA copy number and genome editing activity (12). However, current LVLPs can only package *Cas9* mRNA; thus, sgRNAs have to be delivered separately by other methods. Therefore, an efficient and transient gRNA delivery or Cas9/sgRNA co-delivery method is desirable.

Recently, retrovirus-like particles were explored to deliver *SpCas9* mRNA and single guide RNA (sgRNA) (15). However, adding *MS2* aptamer at various locations of SpCas9 sgRNA decreased nuclease activity of the RNP by 50% (15,16). In addition, *MS2*-containing sgRNAs showed little activity when packaged alone in retrovirus-like particles (15). Thus, it remains unknown why co-packaging *Cas9* mRNA and sgRNA preserves the functions of sgRNA in that study.

It was reported that Cas9 protein was necessary for the stability of sgRNA in cells (17). Here, we explore the possibility of protecting the stability of sgRNA with Cas9 protein and packaging Cas9/sgRNA RNP into lentivirus-like particles via the interactions between ABP and aptamer-modified sgRNA, as well as sgRNA and Cas9. In order to package Cas9 RNP via aptamer/ABP interactions, two requirements have to be met: (i) the aptamer-modified sgRNA should have nuclease activity after forming RNP; (ii) the RNP should be able to be packaged into the lentiviral capsids and survive the post-infection intracellular trafficking. By optimizing the locations and aptamers to be inserted in sgRNA scaffold for most efficient Cas9 RNP encapsulation, we found that Cas9 RNP can be efficiently packaged into lentiviral capsids for efficient ‘hit and run’ gene editing.

## MATERIALS AND METHODS

### Plasmids

pMD2.G (Addgene #12259), psPAX2-D64V (Addgene #63586), pCSII-EF-miRFP709-hCdt(1/100) (Addgene #80007) and pX601-AAV-CMV::NLS-SaCas9-NLS-3xHA-bGHpA;U6::BsaI-sgRNA (Addgene #61591) were purchased from Addgene and have been described previously. Plasmids generated by this group were either described recently (12) or described in Supplementary Table S1. Some of the plasmids will be made available through Addgene. Gene synthesis was done by GenScript Inc. All constructs generated were confirmed by Sanger sequencing. Sequence information for primers, oligos and synthesized DNA fragments is in Supplementary Table S2.

### GFP reporter assays for gene editing activities

The EGFP reporter cells (HEK293T derived) had been described previously (18) and were used to detect gene editing activity of SaCas9/human β-hemoglobin (*HBB*) *sgRNA1* or SaCas9/*IL2RG-sgRNA1* on the inserted target sequences in the GFP-reporter cassette. The GFP-reporter cells expressed no EGFP due to the disruption of the EGFP reading frame by the insertion of the *HBB* sickle mutation and *IL2RG* target sequences between the start codon and the second codon of EGFP coding sequence. Indels formed after gene editing may restore the EGFP reading frame, resulting in EGFP expression. GFP-positive cells were analyzed by fluorescence microscopy or flow cytometry (BD Biosciences, Accuri C6) as described (12).

### AAV6 virus production and transduction

Adeno-associated virus vectors expressing SaCas9 (19) or *HBB sgRNA1* were made from the AAV vector pSaCas9 (expresses SaCas9) or pSaCas9-*HBB*-sgRNA1 (expresses *HBB* sgRNA1 and contains donor template for homologous recombination to change the wild-type *HBB* gene to the Sickle mutation), respectively. AAV serotype 6 (AAV6) production and quantification were performed by Virovek, Inc. (Hayward, CA). AAV6 transduction was performed in serum-free medium or OPTI-MEM at a titer of 10^3^–10^4^ virus genome/cell. Twenty-four hours after transduction, the cells were returned to serum-containing growth medium.

### Lentiviral vector and LVLP production

Lentiviral vector plasmid pCK002-*HBB*-*sgRNA1* expressing both *SaCas9* and *HBB sgRNA1* was used to produce integration-competent lentiviral vector (packaged by packaging plasmid pspAX2) and integration-defective lentiviral (IDLV) vector (packaged by packaging plasmid pspAX2-D64V) as described (12,18). *SaCas9* mRNA LVLP production was described previously (12). To produce Cas9 RNP LVLPs, 13 million HEK293T cells were cultured in 15-cm dish with 15 ml Opti-MEM. About 16 μg of ABP-modified packaging plasmid pspAX2-D64V-NC-ABP [ABP could be MCP (*MS2*coat protein, binding to *MS2*), PCP (*PP7*coat protein, binding to *PP7*), λ N22 peptide (binding to *BoxB*) or COM (binding to com)], 6 μg envelope plasmid (pMD2.G), and 16 μg plasmid DNA co-expressing SaCas9 and the aptamer-modified sgRNA (see Supplementary Figure S3 for plasmid information) were mixed in 1 ml Opti-MEM. About 76 μl of 1 mg/ml polyethylenimine (PEI, Polysciences Inc.) was mixed in 1 ml Opti-MEM. The DNA mixture and the PEI mixture were then mixed and incubated at room temperature for 15 min. The DNA/PEI mixture was then added to the cells in Opti-MEM. Twenty-four hours after transfection, the medium was changed into 15 ml Opti-MEM and the Cas9 RNP LVLPs were collected 48 and 72 h after transfection. The supernatant was spun for 10 min at 500 *g* to remove cell debris before further processing described below.

### Concentrating lentivirus and LVLPs

The LVLP-containing supernatant was concentrated with the KR2i TFF System (KrosFlo® Research 2i Tangential Flow Filtration System) (Spectrum Lab, Cat. No. SYR2-U20) using the concentration-diafiltration-concentration mode. Briefly, 150–300 ml supernatant was first concentrated to about 50 ml, diafiltrated with 500 ml to 1000 ml PBS, and finally concentrated to about 8 ml. The hollow fiber filter modules were made from modified polyethersulfone, with a molecular weight cut-off of 500 kDa. The flow rate and the pressure limit were 80 ml/min and 8 psi for the filter module D02-E500–05-N, and 10 ml/min and 5 psi for the filter module C02-E500–05-N.

### Lentiviral vector and LVLP quantification

Viral titer was determined by p24 based ELISA (Cell Biolabs, QuickTiter™ Lentivirus Titer Kit Catalog Number VPK-107). When un-concentrated samples were assayed, the viral particles were precipitated according to the manufacturer’s instructions so that the soluble p24 protein was not detected.

### Western blotting analysis of viral proteins from lentivirus and LVLPs

Concentrated lentivirus or LVLPs (400 ng p24 by ELISA) were lysed in 60 μl of 1× Laemmli sample buffer. The proteins in each sample were separated on SDS-PAGE gels and analyzed by western blotting. The antibodies used include mouse monoclonal anti-SaCas9 antibody (Millipore Sigma, MAB131872, clone 6F7, 1:1000), rabbit polyclonal HIV1 p17 antibody for matrix protein (MA, ThermoFisher Scientific, Cat No. PA1–4954, 1:1000), rabbit polyclonal HIV1 p15 antibody for nucleocapsid protein (NC, Abcam, Cat No. ab66951, 1:1000) and p24 mouse monoclonal antibody for capsid protein (CA, Cell Biolabs, Cat No. 310810, 1:1000). HRP conjugated anti-Mouse IgG (H+L) (ThermoFisher Scientific, Cat No. 31430, 1:5000) and anti-Rabbit IgG (H+L) (Cat No. 31460, 1:5000) secondary antibodies were used in western blotting. Cas9 RNP standards were from BioVision Incorporation (Cat# M1280–50). Chemiluminescent reagents (Pierce) were used to visualize the protein signals under the LAS-3000 system (Fujifilm). Densitometry (NIH ImageJ) was used to quantitate protein amount.

### RNA isolation from lentivirus or LVLPs and RT-qPCR analysis

A miRNeasy Mini Kit (QIAGEN Cat No. 217004) was used to isolate RNA from concentrated lentivirus or LVLPs. The QuantiTect Reverse Transcription Kit (QIAGEN) was used to reverse-transcribed the RNA to cDNA. For sgRNA reverse transcription, 0.6 μl random primers provided in the kit and 0.4 μl sgRNA-specific primer (sgRNA-R2, 20 μM) were used for reverse transcription. Custom designed Hydrolysis probes specific for *SaCas9* and *EGFP* (ThermoFisher Scientific) were used in qPCR, together with TaqMan Universal PCR Master Mix (ThermoFisher Scientific). For *HBB sgRNA1* and *HBB sgRNA1^Tetra-com^* detection, sgRNA-F1 and sgRNA-R3 were used as primers in SybrGreen based RT-qPCR. PCR was run on an ABI 7500 instrument. Primer information was included in Supplementary Table S2.

### Removing membrane from LVLP capsids

Lentivirus-like particles were transiently treated with 0.5% Triton X-100 following a published procedure (20). Briefly, LVLPs were centrifuged with a Sorvall T-890 rotor (2 h at 120 000 *g*) through step gradients containing a 1 ml layer of 10% sucrose in STE [100 mM NaCl, 50 mM Tris/HCl (pH 7.5), 1 mM EDTA] with or without 0.5% Triton X-100, and a cushion of 2 ml 20% sucrose in STE solution. Pelleted viral particles were directly lysed for western blotting or RT-qPCR analysis.

### Lentiviral vector and LVLP transduction

Concentrated lentiviral vectors or LVLPs (equivalent to 10–300 ng p24 protein) were added to 2.5 × 10^4^ cells grown in 24-well plates, with 8 μg/ml polybrene. Un-concentrated virus containing supernatant was diluted with fresh medium at a 1:1 ratio to transduce cells. The cells were incubated with the particle containing medium for 12 to 24 h, after which normal medium was replaced after transduction.

### Transmission electron microscopy

Transmission electron microscopy was performed at the Cellular Imaging Shared Resource of Wake Forest Baptist Health Center (Winston-Salem, NC). GFP-lentiviral vector and RNP LVLPs concentrated by TFF system (about 1 ng/μl p24) were stained with uranyl acetate. The particles were absorbed on plain carbon grids, dried and observed under a FEI Tecnai G2 30 electron microscope (FEI, Hillsboro, OR). The diameters of the particles were measured with NIH ImageJ software (Version 1.49).

### Gene editing in human cells

For gene editing with Cas9 expressed from AAV serotype 6, the SaCas9 expressing AAV6 and the *HBB sgRNA1* expressing AAV6 were co-transduced into GFP reporter cells. For gene editing with lentiviral vector (LV) or IDLV (integration-defective lentiviral vector, packaged with packaging plasmids containing a D64V mutation in the integrase) expressing both SaCas9 and *HBB sgRNA1*, virus equivalent to 10–300 ng p24 was used to transduce 2.5 × 10^4^ cells in 24-well plates. For gene editing with *SaCas9* mRNA LVLPs, various amounts of *SaCas9* mRNA LVLPs (quantified by p24) were co-transduced into HEK293T cells or the GFP reporter cells with an IDLV-expressing *HBB sgRNA1*. For transduction with Cas9 RNP LVLPs, various amounts of Cas9 RNP LVLPs were transduced into human cells. About 48–72 h after transduction, gene editing activity was analyzed by GFP-reporter assay or next-generation sequencing.

To examine gene editing in human lymphoblastoid cells immortalized by Epstein–Barr virus transformation, human lymphoblastoid cell lines were purchased from Corrie Institute (GM16265, with Sickle cell mutation; ID00085, with mutation in *IL2RG* gene). The lymphoblasts were cultured in RPMI 1640 with 2 mmol/l L-glutamine and 15% fetal bovine serum at 37°C under 5% carbon dioxide. For LVLP and IDLV transduction, 2 × 10^5^ cells were added to 0.5 ml RPMI growth medium. Then, Cas9 RNP LVLPs were added to the cells. Polybrene was added in the medium to a final concentration of 8 μg/ml. Fresh medium was replaced 24 h after transduction. The cells were collected 72 h after transduction for DNA analysis by next-generation sequencing.

### Next-generation sequencing and data analysis

The endogenous *HBB* target sequence and *IL2RG* target sequence, and the *HBB* and *IL2RG* target sequence in the integrated GFP-expression cassette were amplified for sequencing analysis. A nested PCR strategy was used to amplify the endogenous *HBB* target sequence to avoid amplifying the sequence from the viral vector template. First, primers HBB-1849F and HBB-5277R were used to amplify the 3.4 kb region from the *HBB* gene locus. These two primers cannot amplify sequences from the templates in the viral vectors. Then HBB-F2 and HBB-R2 primers were used to amplify the target DNA for sequencing (Supplementary Table S2). To amplify the endogenous *IL2RG* target sequence, primers IL2RG-1029F and IL2RG-3301R were used to amplify the target region from the treated cells (unable to amplify sequences from the templates in the viral vectors), then primers IL2RG-F1 and IL2RG-3301R were used to amplify the target DNA from the first PCR product for sequencing. To amplify the *HBB* target sequence from the integrated EGFP reporter for sequencing, Reporter-F and Reporter-R1 primers were used. The proofreading HotStart® ReadyMix from KAPA Biosystems (Wilmington, MA) was used for PCR. The purified PCR products were shipped to Genewiz Inc. (Morrisville, NC) to perform next-generation sequencing (Amplicon EZ). Usually, 50 000 reads/amplicon were obtained.

Analysis of Insertions and deletions (Indel) was done with the online Cas-Analyzer software (21). The total Indel rate was the difference between 1 and the percentage of readings without mutation. The top 8–10 most frequently observed readings were presented.

### Monitoring the speed of GFP-positive cell emergence

About 2.5 × 10^4^ GFP-reporter cells were seeded in 24-well plates and transduced with 50 ng p24 of *IL2RG* RNP LVLPs, or co-transduced with 100 ng p24 of *Cas9* mRNA LVLPs and 100 ng p24 of IDLV-expressing *IL2RG sgRNA1*. The cells were then incubated in the IncuCyte S3 system (Essen BioScience, Inc. Ann Arbor, Michigan) for timed GFP fluorescence scanning. Two wells from each treatment and nine spots from each well were scanned. The scanning started right after transduction and the cells were scanned once every 2 for 48 h. The GFP-positive rate of each image was calculated by dividing the GFP-positive area by the phase area (area occupied by cells) in that image.

### Statistical analysis

GraphPad Prism software (version 5.0) was used for statistical analyses. *T*-tests were used to compare the averages of two groups. Analysis of variance (ANOVA) was performed followed by Tukey post-hoc tests to analyze data from more than two groups. Bonferroni post-hoc tests were performed following ANOVA in cases of two factors. *P* < 0.05 was regarded as statistically significant.

## RESULTS

### Replacing the sgRNA Tetraloop with aptamer best preserved the Cas9/sgRNA RNP activity

**Figure 1.**
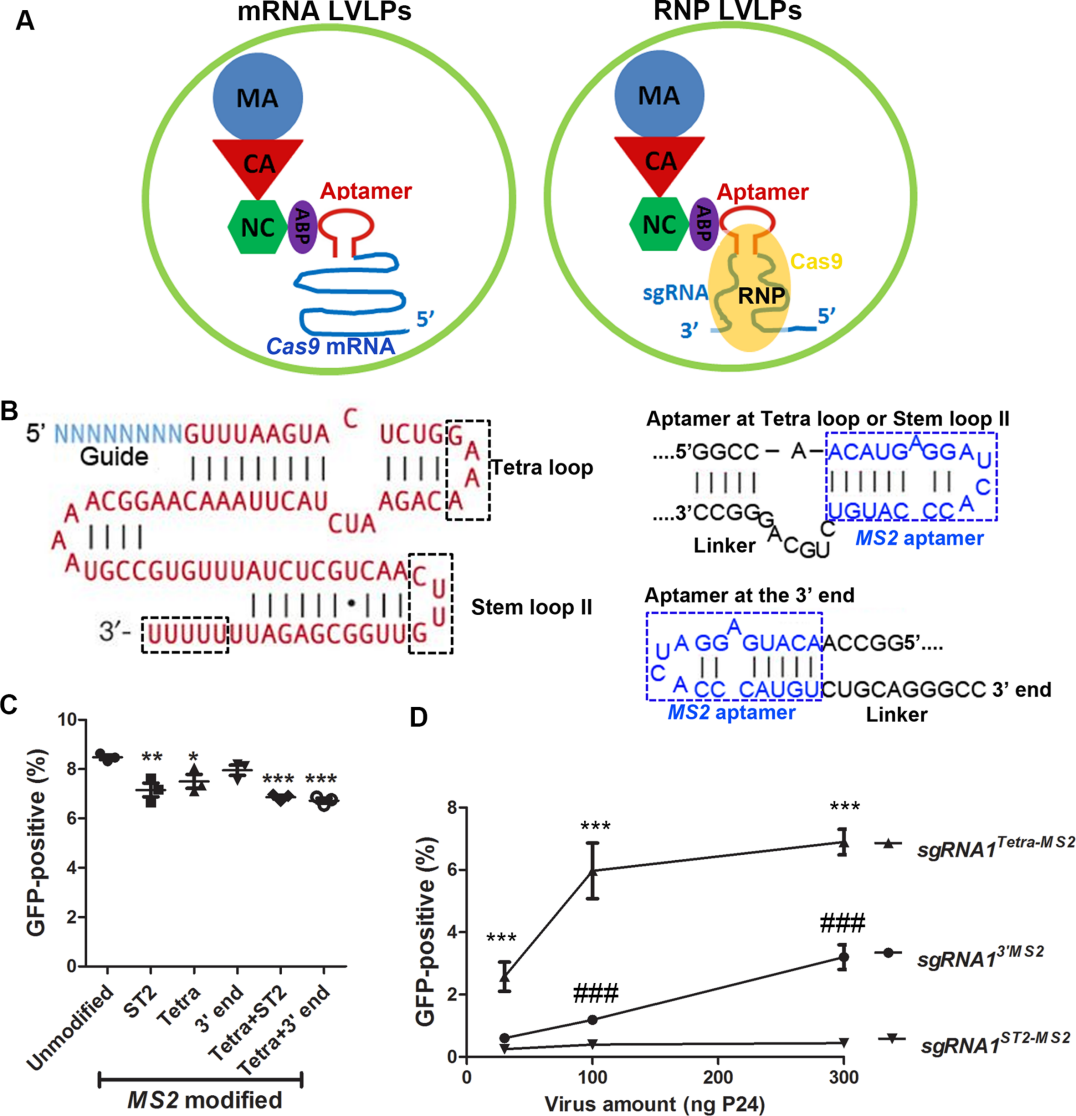
Packaging sgRNA in LVLPs. (**A**) Diagram illustrating the difference between *Cas9* mRNA LVLP (left) and Cas9/sgRNA RNP LVLP (right) as immature virion. Only one GAG precursor and one mRNA or RNP is shown for simplicity. ABP may bind to aptamer as dimers. Envelope proteins are not shown. (**B**) Locations for *MS2* aptamer insertion in sgRNA scaffold. The original sgRNA is shown on the left. The ‘N’s indicate guide sequence. The three locations tested for *MS2* aptamer insertion are indicated by the dashed black boxes. The inserted sequences are shown on the right. The blue letters in dashed blue boxes are the *MS2* aptamers and the black letters are added linkers. Complementary ribonucleotides are indicated by vertical lines and atypical base-pairings are indicated by dots. (**C**) Effects of *MS2* aptamer position on gene editing activity of the RNP with the modified sgRNA. Plasmid DNA co-expressing *SaCas9* mRNA and various modified *HBB sgRNA1* were transfected into the GFP-reporter cells and the GFP-positive percentage was determined by flow cytometry. Each data point indicates one independent experiment. *, ** and *** indicate *P* < 0.05, *P* < 0.01 and *P* < 0.001 (Tukey’s Multiple Comparison Test following ANOVA) when compared with unmodified *HBB sgRNA1*. (**D**) Effects of *MS2* aptamer position on gene editing activity of modified sgRNA packaged in LVLPs. Indicated amounts of LVLPs containing *MS2*-modified *HBB sgRNA1* and SaCas9 protein (or mRNA) were used to transduce 2.5 × 10^4^ GFP reporter cells and the GFP-positive percentages were determined by flow cytometry. Each data point is the average of three replicates. ***, *P* < 0.001 when *HBB sgRNA1*^Tetra^*^MS2^* was compared with *HBB sgRNA1*^3′^*^MS2^*; ###, *P* < 0.001 when *HBB sgRNA1*^3′^*^MS2^* was compared with *HBB sgRNA1^ST2MS2^*.

Several studies have shown that sgRNA is unstable without Cas9 protein protection (15,17); we thus tested the idea of packaging Cas9/sgRNA RNP into lentivirus-like particles, hoping that sgRNA can be protected by Cas9 protein. The overall strategy is to incorporate ABP into lentivirus-like particles via fusing ABP with the lentiviral nucleocapsid protein (NC) as we reported recently (12), and add the corresponding aptamer into sgRNA, which complexes with Cas9 protein and forms RNP during lentiviral capsid assembly. The RNP will be packaged into the lentiviral capsids via the specific aptamer/ABP interaction (Figure 1A). After escaping from the endosomes, the RNP is released into the cytoplasm following capsid uncoating, and the RNP complex will then enter the nucleus to perform gene editing.

In order for this strategy to work, it is necessary to find a location in the sgRNA scaffold best tolerating aptamer insertion and preserving the nuclease activity after complexing with Cas9. We started with *MS2* aptamer since it mediated efficient *Cas9* mRNA packaging in our recent study (12). Three locations were tested: inserting *MS2* into the Stem loop 2 (ST2), replacing the Tetraloop with *MS2* and adding *MS2* after the 3′ end of the sgRNA (Figure 1B). When the plasmid DNAs co-expressing SaCas9 and the modified sgRNAs targeting *HBB sgRNA1* were transfected into the GFP-reporter cells, Indels in *HBB sgRNA1* target sequence may restore GFP expression (18). We found that adding one *MS2* at any of the three locations preserved gRNA activity in transfection experiments, although replacing the Tetraloop or the ST2 loop slightly decreased the percentage of GFP-positive reporter cells (Figure 1C). We also tested addition of two *MS2* aptamers, one replacing the Tetraloop, and the other one at either the ST2 loop or 3′ end. In both cases, the GFP-positive percentages were decreased consistently, agreeing with observations that more than one copy of *MS2* decrease RNA stability (12,22). Thus, we decided to use one copy of aptamer in further experiments.

We then tested whether these *MS2*-modified sgRNAs could be packaged and delivered by LVLPs. We co-expressed SaCas9 protein and *MS2-*modified *HBB sgRNA1* during LVLP preparation, and transduced the LVLPs into our GFP reporter cells. Flow cytometry analysis found that LVLPs containing *HBB sgRNA1* with *MS2* at the Tetraloop (*HBB sgRNA1*^Tetra^*^MS2^*) gave the most GFP-positive cells, LVLPs containing *HBB sgRNA1* with *MS2* at the 3′ end (*HBB sgRNA1*^3′^*^MS2^*) followed. In contrast to transfection experiments, where *MS2* at Stem loop II (*HBB sgRNA1^ST2^ ^MS2^*) was active, *HBB sgRNA1^ST2^ ^MS2^* LVLPs showed hardly any gene editing activities (Figure 1D), suggesting that either *HBB sgRNA1^ST2^ ^MS2^* could not be packaged or they could not survive the post-transduction process. Since *MS2*-free *SaCas9* mRNA could also be packaged into LVLPs to some degree (12), at this point we were unsure whether the gene editing activity was from co-packaged *Cas9* mRNA and sgRNA, packaged Cas9/sgRNA RNP or both. This question will be addressed in subsequent experiments; nevertheless the available data showed that Tetraloop is the best position for aptamer addition.

### *com*/COM is the best aptamer/ABP pair for sgRNA packaging

**Figure 2.**
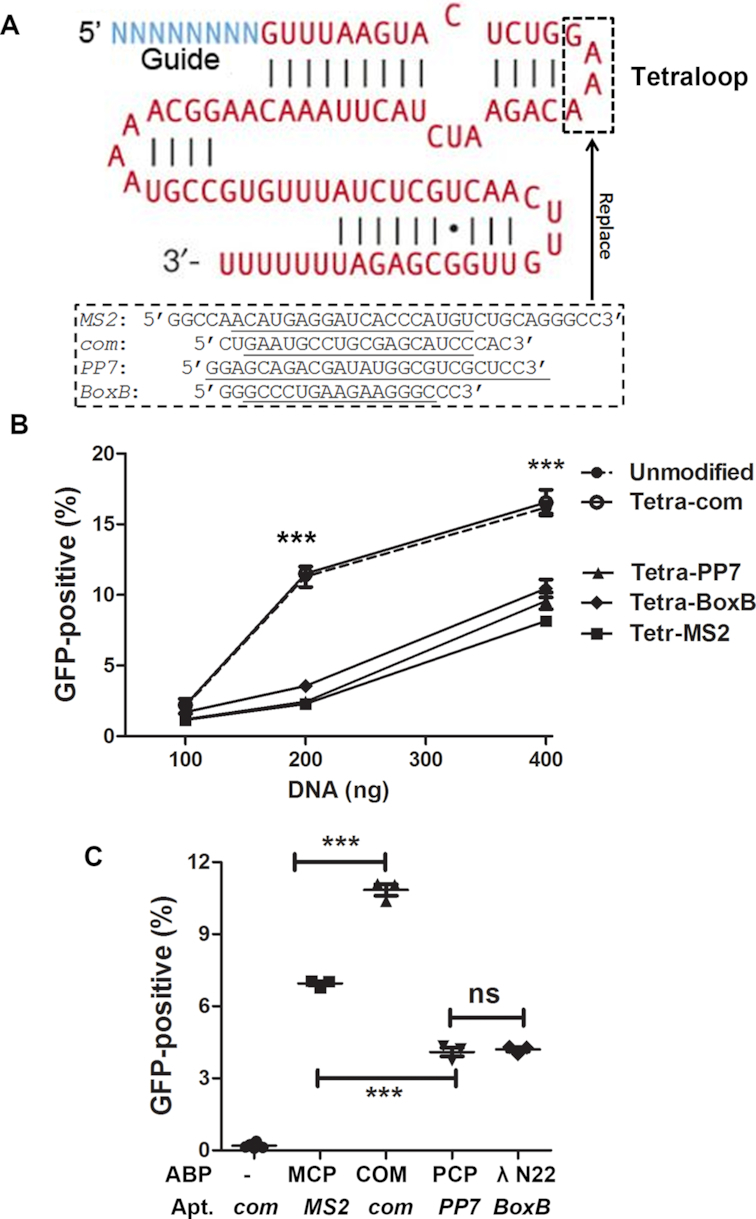
Comparing aptamer/ABP pairs for packaging sgRNA in LVLPs. (**A**) Replacing the Tetraloop with different aptamers for sgRNA packaging. The boxed Tetra loop (GAAA) sequence was replaced with sequences containing various aptamers (underlined) with or without linkers (not underlined). (**B**) Comparing gene editing activities after the Tetraloop was replaced by different aptamers. Various amounts of plasmid DNA co-expressing SaCas9 and aptamer-modified *HBB sgRNA1* was transfected into 1.25 × 10^5^ GFP reporter cells. Forty-eight hours after transfection the GFP-positive cells were analyzed by flow cytometry. Each point was the average of three replicates. *** indicates *P* < 0.001 when sgRNA without modification or with com modification was compared with *PP7, BoxB* or *MS2* modified sgRNAs (Bonferroni post-tests following ANOVA). (**C**) Comparison of gene editing activities of LVLPs made by different aptamer/ABP pairs. Four hundred microliters of un-concentrated LVLPs were used to transduce 2.5 × 10^4^ GFP reporter cells, and the GFP-positive rate was determined by flow cytometry. Each point indicates one independent assay. *** indicates *P* < 0.001 between the indicated pairs in Tukey’s Multiple Comparison Test following ANOVA analysis; ns, not significant.

Knowing that replacing the Tetraloop with aptamers performed the best, we continued to search for the aptamers to be used to replace the Tetraloop for most efficient gene editing. Four aptamers, *MS2* (23), *PP7* (24), *BoxB* (25) and *com* (26), have been used in sgRNAs for target-binding purposes (22,27–29). We compared the activities of *HBB sgRNA1* with various aptamer replacing the Tetraloop (Figure 2A). When plasmid DNA co-expressing SaCas9 and various aptamer-modified *HBB sgRNA1* were transfected into GFP reporter cells, replacing Tetraloop with *com* aptamer generated the same rates of GFP-positive cells as the unmodified sgRNA, while replacing Tetraloop with the other three aptamers reduced the GFP-positive rates (Figure 2B).

We then tested whether the *HBB sgRNA1* with different aptamers could be packaged into LVLPs. For this purpose, we used MCP (*MS2*coat protein, binding to *MS2*), PCP (*PP7*coat protein, binding to *PP7*), λ N22 peptide and COM modified packaging plasmids, where ABPs were inserted after the second zinc finger domain of nucleocapsid protein (NC) as described recently (12), to make LVLPs containing RNPs in which Cas9 complexed with various modified *HBB sgRNA1*. We then transduced these LVLPs into GFP-reporter cells. Flow cytometry showed that LVLPs generated by *com*/COM pair had the most GFP-positive cells, those generated by *MS2/*MCP followed, *PP7*/PCP and *BoxB*/λ N22 generated LVLPs had the lowest activity (Figure 2C). GFP-positive cells could only be observed when these LVLPs were used to transduce GFP-reporter cells but not HEK293T cells (Supplementary Figure S1), excluding possible contamination of GFP-expressing DNA or viral vector.

Since *com*/COM combination obtained the best results, we examined whether COM-modification of the packaging plasmid impairs particle assembly. We noticed a marginal 11% decrease in particle assembly efficiency compared with the unmodified packaging plasmid (Supplementary Figure S2). This slight decrease should not prevent us from producing sufficient particles. Through these experiments we found the location (Tetraloop) and the aptamer/ABP pair (*com*/COM) for the most efficient packaging of Cas9 RNPs (or Cas9 mRNA and sgRNA) into lentiviral capsids.

### RNPs in the LVLPs accounted for the observed gene editing activity

**Figure 3.**
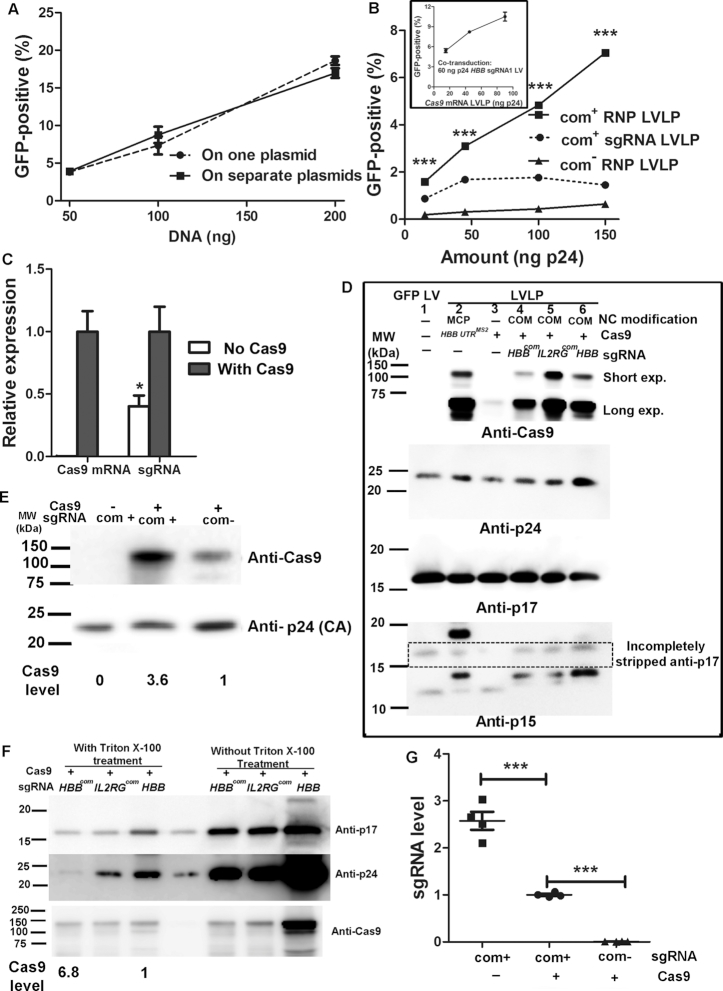
RNP accounted for the gene editing activity. (**A**) *HBB sgRNA1* expressed from transfected plasmid DNA was functional. In co-transfection experiments, the DNA amount indicated each of the Cas9 expressing- and the sgRNA expressing-plasmid DNA. Each point was the average of three replicates. (**B**) Importance of Cas9 co-packaging and com-aptamer modification of sgRNA on gene editing activity of the LVLPs. LVLPs packaged in the absence of Cas9 expression were inactive when co-transduced into GFP-reporter cells with functional *Cas9-HBB-3′UTR^MS2^* mRNA LVLPs. About 2.5 × 10^4^ GFP-reporter cells were transduced with indicated particles. GFP-positive cells were determined by flow cytometry 48 h after transduction. Each point is the average of three replicates. *** indicates *P* < 0.001 when GFP-positive rates of cells treated with com^+^ RNP LVLPs were compared with cells treated with similar amounts of other particles (Bonferroni posttests following ANOVA). (**C**) Co-expressing Cas9 increased *HBB sgRNA1* level. Plasmids expressing only *HBB sgRNA1^Tetra-com^* (200 ng) and only SaCas9 (200 ng) were transfected into HEK293T cells alone or together, and the sgRNA level was compared by RT-qPCR. A GFP-expressing plasmid (50 ng) was co-transfected so that GFP expression could be used to normalize transfection efficiency. Total plasmid DNA was brought to 450 ng by pCDNA3 plasmid DNA. * indicates *P* < 0.05 when sgRNA level without Cas9 co-expression was compared with that of with Cas9 co-expression. (**D**) Western blotting analysis of Cas9 protein in isolated lentiviral vectors and LVLPs. About 200 ng p24 of GFP lentivirus (lane 1), NC-MCP modified *Cas9-HBB-3′ UTR^MS2^* LVLPs (without sgRNA, lane 2), NC-unmodified *Cas9^MS2^* LVLPs (without sgRNA, lane 3), NC-COM modified Cas9/*HBB sgRNA1^tetra-com^* LVLPs (lane 4), NC-COM modified Cas9/*IL2RG sgRNA1^tetra-com^* LVLPs (lane 5) and NC-COM modified Cas9/*HBB sgRNA1* LVLPs (sgRNA without Tetra-com aptamer, lane 6) were loaded. (**E**) Tetra-com modification of sgRNA increased the Cas9 protein content in LVLPs. (**F**) Cas9 proteins in LVLPs with com-modified sgRNA are more detergent-resistant than Cas9 proteins in LVLPs with unmodified sgRNA. The same amount of starting LVLPs (200 ng of p24) was centrifuged through 1 ml of 10% sucrose with or without 0.5% Triton X-100. For panels (E) and (F), Cas9 level was normalized by CA protein, based on dosimetry analysis (IMAGE J). (**G**) Packaging of sgRNA in LVLPs is com-aptamer but not Cas9 protein dependent. See panel (E) for evidence of similar particle input (CA) for RNA isolation. Each point indicates one repeat. *** indicates *P* < 0.001 between the indicated pairs in Tukey’s Multiple Comparison Test following ANOVA analysis.

Although designed to package RNPs, considering our previous observation that aptamer-free *SaCas9* mRNA could be packaged for unknown mechanisms (12), the observed gene editing activity could be from the co-packaged *SaCas9* mRNA and sgRNA. To determine whether RNP or *SaCas9* mRNA/sgRNA contributed to the observed gene editing activity, we separated the Cas9 expression cassette from the *HBB sgRNA1^Tetra-com^* expression cassette into two different plasmids (Supplementary Figure S3). When the two plasmids were co-transfected into our GFP reporter cells, they could generate GFP-positive cells as efficiently as transfecting a single plasmid containing both expression cassettes (Figure 3A). However, when *HBB sgRNA1^Tetra-com^* was packaged into LVLPs in the absence of Cas9 expression, these LVLPs had little gene editing activity when co-transduced into GFP-reporter cells with *Cas9* mRNA LVLPs (Figure 3B, dashed lines, com^+^ sgRNA LVLP). *Cas9* mRNA LVLPs were functional and 45 ng p24 of *Cas9* mRNA LVLPs were able to generate 9% GFP^+^ reporter cells when co-transduced into GFP-reporter cells with 60 ng p24 of *HBB* sgRNA1-expressing lentiviral vectors (insert in Figure 3B).

Previously, it was shown that Cas9 protein is needed to protect the stability of sgRNA in cells (17). We confirmed this observation by expressing *HBB sgRNA1^Tetra-com^* alone or co-expressing sgRNA with Cas9. Co-expressing Cas9 significantly increased the expression of *HBB sgRNA1^Tetra-com^* (Figure 3C). After DNA transfection, the sgRNA was constitutively expressed. Whereas after transduction, the sgRNAs had to complete the post-transduction intracellular trafficking and there was no new sgRNA generation. Thus, it is expected that the protective effects of Cas9 protein on sgRNA would be more critical when packaged in LVLPs. The data suggest that sgRNA instability, especially their inability to survive during the post-transduction intracellular trafficking, most likely explains why singly packaged *HBB sgRNA1^Tetra-com^* LVLPs had little activity after co-transducing with *Cas9* mRNA LVLPs (Figure 3B).

These experiments argue against a major contribution from co-packaged *Cas9* mRNA and sgRNA, and argue for the packaging of Cas9 RNPs, since otherwise the unprotected sgRNA would have little activity even if *Cas9* mRNA could efficiently produce Cas9 protein after transduction. This would predict the existence of Cas9 protein in the LVLP particles. To examine the presence of Cas9 protein in the LVLPs, we did western blotting analyses on the concentrated LVLPs (Figure 3D).

Viral proteins Matrix (MA, p17) and Capsid (CA, p24) were detected as the expected size, indicating that the processing of MA and CA were unaffected. Detection of p15, from which Nucleoprotein (NC) was processed, showed a band between 10 and 15 kDa in GFP lentivirus (lane 1) and in LVLPs generated with the unmodified packaging plasmid (lane 3). In LVLPs generated by NC-MCP modified packaging plasmid (lane 2), a strong band between 15 and 20 kDa was detected, which was slightly smaller than the expected 21.8 kDa NC-MCP fusion protein. While in LVLPs generated by NC-COM modified packaging plasmid (lanes 4–6), a band slightly smaller than 15 kDa was detected, which was slightly smaller than the expected 18.7 kDa of the NC-COM fusion protein. Anti-p15 detected bands slightly smaller than expected in all samples (including GFP lentivirus), which could be caused by our SDS-PAGE system or by partial degradation of the p15 or p15-fusion proteins.

We then examined the presence of Cas9 protein in these particles. As expected, Cas9 protein was not detected in GFP-expressing lentiviral vectors (Figure 3D, lane 1). However, it was detected in all types of LVLPs produced in cells with Cas9-expression, no matter whether there was sgRNA (lane 3, observed after longer exposure), or whether the sgRNA was modified by *com* aptamer (Figure 3D, lane 6). The detection of Cas9 proteins in the LVLPs suggested that Cas9 protein could contribute to the observed gene editing activities. However, Cas9 protein was also detected in LVLPs without *com*, it remained to be determined whether *com* has any function in Cas9 packaging, and why LVLPs with Cas9 and *com*^−^ sgRNA had little gene editing activity (Figure 3B, *com*^−^ RNP LVLPs).

### *com*-modification of sgRNA was necessary for efficiently packaging Cas9/sgRNA RNPs into the core capsid of LVLPs

We noticed that in Figure 3D, although we added similar amount of particles based on p24 ELISA, the p24 and p15 signals in western blotting differed greatly between *com^+^* and *com^−^* RNP LVLPs (lane 4 and 6 in Figure 3D). We reasoned that this was likely caused by different reactivity of anti-p24 antibody to p24 (CA) in different types of capsids, and this large difference in p24 input made it difficult for us to compare the amount of Cas9 protein/particle between different LVLPs.

To examine whether *com* could increase Cas9 protein packaging in LVLPs, we adjusted the input of *com^+^* and *com^−^* RNP LVLPs (samples in lane 4 and lane 6 of Figure 3D) so that the p24 inputs were similar. After input adjustment, we found that the *com^+^* RNP LVLPs contained 2.6-fold more Cas9 protein than *com^−^* RNP LVLPs did (Figure 3E). The data showed that *com* increased the amount of Cas9 protein packaged in LVLPs.

Our LVLPs were concentrated by tangential flow filtration, a process also retaining membranous structures such as exosomes. We reasoned that the Cas9 protein detected in the previous experiments might have two sources: membrane-associated and LVLP capsid-packaged. *com*, if worked, should increase the capsid-packaged Cas9 protein. To distinguish the capsid-packaged Cas9 protein from the membrane-associated Cas9 protein, we transiently treated the LVLP particles with 0.5% Triton X-100 as described previously (20) to eliminate Cas9 proteins associated with membrane vesicles and capsid envelope. We found that Triton X-100 treatment greatly decreased the amount of MA protein (detected by p17 antibody) associated with the capsid envelope in all samples, indicating that the treatment worked (Figure 3F). The CA protein (detected by p24 antibody) was reduced to variant degrees, which could reflect different core capsid stability of different types of particles. However, the amount of Cas9 protein was greatly reduced in *com*^−^ RNP LVLPs, but only slightly reduced in the *com*^+^ RNP LVLPs. *com*^+^ RNP LVLPs had 6.8-fold detergent-resistant Cas9 protein compared to *com*^−^ RNP LVLPs, suggesting that *com* contributed to packaging of 85% (5.8/6.8*100%) of the total Cas9 protein. These data show that *com* modification of sgRNA facilitated the packaging of Cas9 protein in the detergent-resistant capsid core, and more core-protected RNPs correlated with their high gene editing activity.

We also examined the importance of *com*-aptamer on sgRNA packaging by RT-qPCR analysis of sgRNA content in LVLPs. We realize that sgRNAs are short and form secondary structures, thus the efficiency of sgRNA reverse transcription could be limited. RT-qPCR may underestimate sgRNA level in absolute quantification, but may still be suitable for relative expression comparison. Although our PCR primers produced amplicons of different sizes from *HBB sgRNA1* and *HBB sgRNA1^Tetra-com^* due to the presence and absence of the 23 nt *com* aptamer, the primers generated very similar standard curves when variant amounts of plasmid DNA were used as the templates for qPCR (Supplementary Figure S4), demonstrating similar amplification efficiency for the *com*^+^ and *com*^−^ sgRNA sequences. In the absence of Cas9 protein, *com*^+^*HBB sgRNA1* was packaged into LVLPs 1.5 times more efficiently than *com*^+^*HBB sgRNA1* in the presence of Cas9 protein. However, in the presence of Cas9 protein, com^−^*HBB sgRNA1* was packaged into LVLPs 50-fold less efficient than *com*^+^*HBB sgRNA1* (Figure 3G). These data showed that the packaging of sgRNA was *com* but not Cas9 protein dependent. Cas9 protein slightly decreased the amount of *com^+^* sgRNA packaged, most likely due to the negative effects of Cas9/sgRNA association on com/COM interaction. Thus, our data showed that the packaging of Cas9 protein into the capsid core is mediated by *com^+^* sgRNA via com/COM interaction. Although Cas9 protein is not needed for packaging sgRNA into the LVLPs, it is needed to protect the sgRNAs during transduction.

Except for experiments described in Figure 3, where sgRNA with and without com-modification were used for comparison, *com*-modified sgRNA was used in all subsequent experiments. Hereafter, RNP was used to indicate com^+^ RNP for simplicity.

### Characterization of Cas9 RNP LVLPs

To get an idea of the amount of Cas9 protein in the LVLPs, we quantified the Cas9 protein amount in LVLPs with commercially purchased Cas9 RNPs by western blotting. Two closely located Cas9 bands were observed in RNP LVLPs (Supplementary Figure S5), which could be the results of partial protein degradation during sample handling or storage, since only one band was observed in other experiments (Figure 3) using the same batch of particles. Conforming previous data, Triton X-100 treatment only slightly reduced Cas9 protein amount suggesting that majority of the proteins were resistant to Triton X-100 treatment. The Cas9 protein detected in 67 ng p24 of LVLPs (Triton X-100 treated) was about 1 pmol based on purified Cas9 RNP standards (Supplementary Figure S5). This translates into 720 Cas9 molecules per particle according to the estimation that 1 ng p24 of lentiviral vector contains 1.25 × 10^7^ particles. Since about 85% of the Cas9 protein detected in our RNP LVLPs was enriched by *com*, the estimated RNP number per capsid was about 720 × 85% = 612. Theoretically each capsid has 2000–2500 gag precursors (30), equivalent to 2000–2500 NC-COM fusion proteins and 2000–2500 *com*-binding sites. In addition, the cone shaped HIV capsid (100–200 nm long and 45–50 nm wide) (31) is 400- to 800-fold larger than a Cas9 RNP (10 × 10 × 5 nm) (32). Thus, our estimation of about 600 RNPs per particle is reasonable.

The *com^+^* and *com^−^* RNP LVLPs (with *com*-modified or unmodified *HBB* sgRNA1) were examined under transmission electron microscopy. Both types of LVLPs showed similar morphology compared with normal GFP expressing LV (Supplementary Figure S6). com^+^ RNP LVLPs had slightly larger size compared with GFP-LV (com^+^ RNP LVLPs 117 ± 5.9 nm, *n* = 24; GFP LV 96 ± 4.6 nm, *n* = 24; *P* < 0.05), possibly due to NC-COM fusion and packaging of RNPs. *com*^−^ RNP LVLPs also had larger average diameter than GFP-LV but the difference was not statistically significant (109.5 ± 4.6 nm, *n* = 26). During electron microscopy observations, we observed numerous membrane structures in our LV and LVLP preparations, confirming the existence of membrane structures in our LVLP samples.

### RNP LVLPs showed efficient genome editing and decreased off-targets

**Figure 4.**
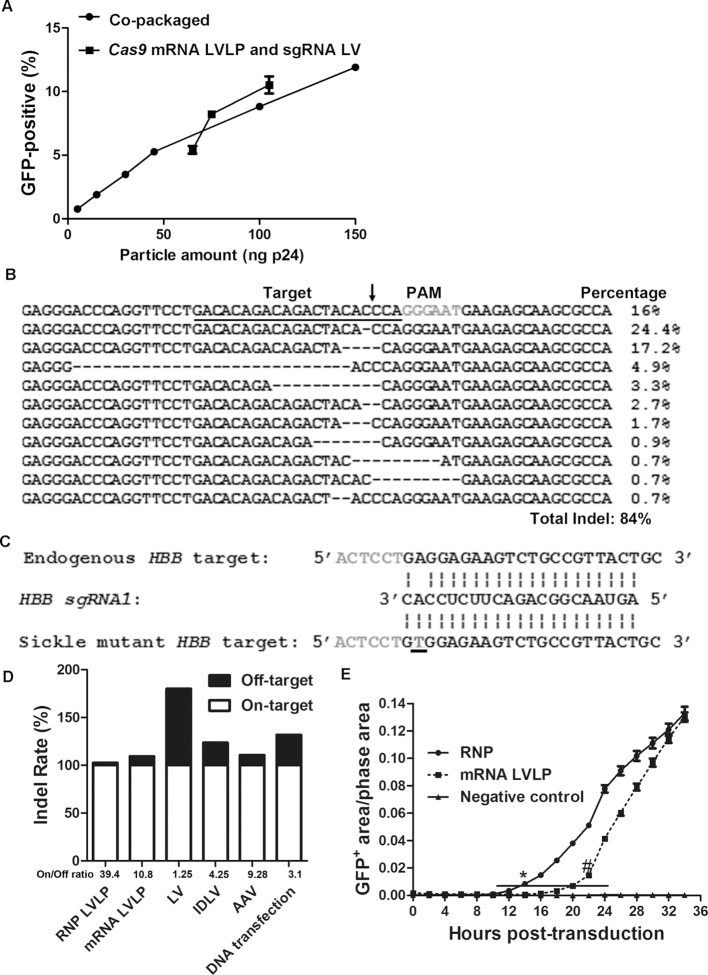
Cas9/sgRNA RNP LVLPs are efficient and specific in gene editing. (**A**) Cas9/sgRNA RNP LVLPs showed comparable gene editing activity as *Cas9* mRNA LVLPs on *HBB* SCD mutant sequence. For *Cas9* mRNA LVLPs, the particle amount was the sum of *Cas9* mRNA LVLPs and 60 ng p24 of *HBB sgRNA1*-expressing LVs. (**B**) Indels generated by Cas9/*IL2RG sgRNA1* RNP LVLPs on the endogenous *IL2RG* target sequence of HEK293T cells. The protospacer adjacent motif (PAM) is in gray and the target sequence is underlined. The predicted cleavage site is indicated by an arrow. The dashed lines indicate deletions. (**C**) Diagram showing the sequence of *HBB sgRNA1*, the Sickle mutant sequence perfectly matching the gRNA, and the endogenous *HBB* sequence with one mismatch with the gRNA. The mutation causing Sickle cell disease is underlined. (**D**) Comparison of on target and off-target Indel rate of Cas9/*HBB sgRNA1* RNP LVLPs with those of other delivery vehicles. The on-target to off-target Indel rate ratios (‘on/off’ ratios) are the results of on-target Indel rates divided by off-target Indel rates. Cells were harvested 72 h after treatment. For cells grown in 24-well plates, 1.25 × 10^5^ and 2.5 × 10^4^ cells were used for transfection and transduction, respectively. For RNP LVLPs, 55 ng p24 were used; for mRNA LVLPs, 45 ng p24 of *Cas9* mRNA LVLPs and 60 ng p24 of *HBB sgRNA1*-expressing IDLV were used; for LV co-expressing Cas9 and *HBB sgRNA1*, 30 ng p24 were used; for IDLV co-expressing Cas9 and *HBB sgRNA1*, 30 ng p24 were used; for Cas9-expressing AAV6 and *HBB sgRNA1*-expressing AAV6, 10^4^ virus genome/cell were used for each virus. On-target Indel rates are all normalized to 100 for comparison. Original data are in Supplementary Table S3. (**E**) Cas9 RNP LVLPs showed faster actions than *Cas9* mRNA LVLPs. About 50 ng p24 of Cas9/*IL2RG sgRNA1* RNP LVLPs, or 100 ng p24 of *Cas9* mRNA LVLPs plus 100 ng p24 of IDLV expressing *IL2RG sgRNA1* were transduced into 2.5 × 10^4^ GFP reporter cells and incubated in IncuCyte for scanning. * indicates the time point from which RNP-treated cells showed significantly higher GFP-positive area/phase area than negative control or mRNA LVLP-treated cells (*P* < 0.05). # indicates the time point from which mRNA LVLP-treated cells showed significantly higher GFP-positive area/phase area than negative control cells (*P* < 0.05). The line shows the time lag in *Cas9* mRNA LVLP treated cells to reach the same level of GFP-positive area/phase area as RNP-treated cells.

We compared the gene editing activities of RNP LVLPs described in this report and the *Cas9* mRNA LVLPs described recently (12). *HBB sgRNA1* RNP LVLPs showed comparable gene editing activities as the *SaCas9* mRNA LVLPs in GFP-reporter assays (Figure 4A).

We further examined the gene editing activities of the RNP LVLPs by next-generation sequencing (NGS) on another target, *IL2RG*, using *IL2RG sgRNA1* described recently (12,18). About 150 ng p24 of *IL2RG sgRNA1* RNP LVLPs were transduced into 2.5 × 10^4^ GFP reporter cells. Seventy-two hours after transduction, the endogenous *IL2RG* region was amplified and subjected to NGS. About 84% Indels were observed in the endogenous *IL2RG* gene (Figure 4B), in contrast to 0.03% Indel rate of negative control cells transduced with AAV6 expressing Cas9 and *HBB* sgRNA1. In our previous study, 30 ng of p24 of *SaCas9* mRNA LVLPs and 60 ng of *IL2RG* sgRNA1 IDLV generated 13% Indels in *IL2RG* of HEK293T cells (12), thus our RNP LVLPs showed better gene editing activity. We also transduced 200 ng of p24 of *IL2RG sgRNA1* RNP LVLPs into 2 × 10^5^ B lymphoblastoids, and detected 18.3% Indels (Supplementary Figure S7). This activity is also better than 100 ng of p24 of *SaCas9* mRNA LVLPs and 100 ng of p24 of *IL2RG* sgRNA1 expressing IDLV, which generated 11% Indels in the same number of B lymphoblastoids (12).

We usually used 100–200 ng p24 of RNP LVLPs to transduce 2.5 × 10^4^ cells. Based on our western blotting data that 67 ng of p24 RNP LVLPs contained 1 pmol RNP; we typically used 0.6–1.2 pmol RNP for 10^4^ cells. The RNP amount used in electroporation of human cells is typically 10–20 pmol RNP for 10^4^ human somatic cells (33). Thus, the RNP dose we used for our RNP LVLPs was over 10 times lower than those used for biochemically purified RNPs.

These data show that the RNP LVLPs were as efficient as or more efficient than our recently reported *Cas9* mRNA LVLPs in gene editing. We reasoned that delivering Cas9 by RNP should offer better control of the amount of Cas9 protein delivered per cell and more transient Cas9 function. Both would help to reduce off-target rates. The predicted nine potential *HBB sgRNA1* off-targets all had very low Indel rates (12), preventing us from comparing different off-target rates between different delivery methods. However, the wild-type *HBB* sequence corresponding to the Sickle cell disease mutation has only 1 nucleotide mismatch with *HBB sgRNA1* (Figure 4C) and detectable off-target Indels could be generated by Cas9/*HBB sgRNA1* (12). We thus compared the on-target Indel rates (in the Sickle cell disease mutation in GFP reporter cassette, shown at the bottom of Figure 4C) and the off-target Indel rates (in the endogenous wild-type *HBB* sequence with 1 nucleotide mismatch to *HBB sgRNA1*, shown on the top of Figure 4C) in GFP-reporter cells. The Cas9/*HBB sgRNA1* was delivered by plasmid transfection, or by lentiviral vector (LV), IDLV or adeno-associated virus (AAV) as described recently (12). We found that RNP LVLP had the highest ratio of on-target to off-target Indel rates, *Cas9* mRNA LVLPs had the second, and both were higher than those of LV, IDLV and AAV (Figure 4D and Supplementary Table S3). Thus, RNP LVLPs showed the best capability of distinguishing on-targets from off-targets.

### RNP LVLPs showed faster action than *Cas9* mRNA LVLPs after transduction

We compared the kinetics of GFP-positive cell emergence after RNP LVLP and *Cas9* mRNA LVLP treatment in GFP reporter assays. For this purpose, we transduced 2.5 × 10^4^ GFP reporter cells with 50 ng of p24 of *IL2RG sgRNA1* RNP LVLPs, or co-transduced GFP reporter cells with 100 ng of p24 of *Cas9* mRNA LVLPs and 100 ng of p24 of IDLVs expressing *IL2RG sgRNA1*. The emergence of GFP-positive cells was monitored every 2 h. We found that GFP-positive cells showed up at least 6 h earlier in the RNP LVLP treated cells than in the mRNA LVLP treated cells (Figure 4E and Supplementary Figure S8). Thirty-four hours after transduction, the GFP-positive rates of the two treatments converged. The data showed that RNP LVLPs have faster actions than mRNA LVLPs plus IDLVs. The reason is most likely because that the RNPs were available for function in the nucleus soon after escaping from the endosome, while the mRNA LVLPs and IDLVs needed more time to express Cas9 protein and sgRNA. The difference in kinetics of actions also indicated that the functional components in RNP LVLPs were different from those in the *Cas9* mRNA LVLPs.

## DISCUSSION

Here, we describe a novel lentiviral capsid based bionanoparticle for Cas9 RNP packaging and delivery. We show that as designed, Cas9 RNPs but not *Cas9* mRNA/sgRNAs were packaged in the LVLPs. These bionanoparticles efficiently delivered RNPs for transient Cas9 expression and efficient genome editing, and showed superior on-target to off-target discrimination capability and faster action after transduction.

Finding the best sgRNA location and aptamer/ABP combination is critical for the success of our strategy. For spCas9 sgRNA, *MS2* aptamer addition at various locations decreased RNP activity by 50% (15,16). Here, we found that replacing the sgRNA Tetraloop with *com* aptamer did not impair sgRNA performance, and believed that several factors may contribute to the better performance of our aptamer modified sgRNA. (i) We used one aptamer instead of two since we observed that one aptamer was the best in packaging *Cas9* mRNA (12) and we and others have found that two copies of aptamer impaired sgRNA performance (22). (ii) We screened multiple sgRNA locations and aptamers. Replacing the Tetraloop sequence with aptamer resulted in the best RNP performance, possibly because the Tetraloop is an artificial sequence linking the CRISPR RNA (crRNA) and the trans-activating crRNA (19,34,35), and has no important contacts with Cas9 protein (35). Aptamer at this location is expected to be more flexible and accessible. Inserting *MS2* at stem loop II of the sgRNA showed the lowest activity, which is consistent with the observation that stem loop II has important structural functions (35).

We found that the performance of different aptamer-modified sgRNAs in transfection experiments correlates with those after they were packaged into LVLPs. This suggests that the functionality of the aptamer-modified sgRNA, rather than the packaging of the sgRNA, is a critical factor dictating their performance. Thus, using *com* aptamer to modify sgRNA is the key for the success of our strategy. In contrast to *MS2, PP7* and *BoxB*, which bind to their respective ABP with a specific secondary RNA structure, the core *com* RNA sequence does not fold into a stable secondary structure. This may explain why replacing the Tetraloop by *com* can better preserve the sgRNA function. In addition, the following facts might also contribute to the better performance of the *com*/COM combination: (i) Compared with *MS2*/MCP and *PP7*/PCP combinations, COM is small (62 AA) while MCP and PCP are both twice long; (ii) PCP and MCP bind aptamer as dimers (36), while COM is likely to bind as monomers (26). Although λ N22 is the smallest, it may not be able to form fully functional structure in the Gag precursor.

We showed that Cas9/sgRNA RNPs contributed to the gene editing activities of the LVLPs. The amounts of Cas9 protein in the capsid core correlated with the gene editing activities of the LVLPs, and the amounts of core-associated Cas9 protein depended on *com*-modification of sgRNA. These data suggest that the packaging of Cas9/sgRNA RNPs was mediated by *com*/COM interaction. Although in the absence of Cas9, *com*-modified sgRNA could be efficiently packaged, they had little activity after transduction, most likely because the naked sgRNA could not survive the post-transduction intracellular trafficking. Thus in this system, Cas9 protein and *com*-modified sgRNA are both necessary, the latter enables the packaging of the former and the former protects the latter during transduction.

Although Cas9 RNPs can be biochemically produced and delivered by physical or chemical methods (6–9), our lentiviral capsid based RNP bionanoparticle system has advantages of easy preparation and efficient delivery, since it uses the active cellular entrance pathway of normal lentiviral vectors. Compared with RNPs formed by *Escherichia coli* expressed Cas9, we used 10-fold less LVLP-packaged RNPs (0.6–1.2 pmol RNP/10^4^ cells for RNP LVLPs versus 10–20 pmol/10^4^ cells for purified RNPs) to achieve editing efficiencies of >80%. Although lentiviral capsid has been used to deliver zinc finger nuclease/TALEN (37) or Cas9 protein (11), these particles have low production efficiency and moderate gene editing activities, since the nucleases were incorporated into the particles via fusion with one of the viral structural proteins. We used a completely different mechanism to package the RNP complex, through the specific interaction between aptamer, which is included in the sgRNA scaffold, and ABP, which is fused with lentiviral NC protein. In our system, the Cas9 protein packaged is identical to the one commonly used and is thus fully functional.

A retrovirus-like particle system was described recently to co-packaged *SpCas9* mRNA and sgRNA (15). Their aptamer incorporated sgRNA showed 50% decreased nuclease activity complexing with Cas9, whereas our *com*-incorporated sgRNA showed similar activity as the unmodified sgRNA. We observed that inserting COM after the second zinc finger motif preserved 90% of normal particle assembly efficiency. In the retrovirus-like particle system (15), the NC protein, which plays important role in viral particle assembly (38), was replaced by two MCP proteins. The effect of this treatment on particle assembly is unknown and this was not addressed in that study. Importantly, it is unknown whether *Cas9* mRNA/sgRNA or Cas9/sgRNA RNP contributed more to the gene editing activity observed in those retrovirus-like particles.

Compared with our recently described *Cas9* mRNA LVLPs (12), the RNP LVLPs have multiple advantages: (i) they provide both components needed for gene editing in one particle, which simplifies nuclease production and improves gene editing activity since cells will always receive the fully functional complex; (ii) since RNPs instead of mRNAs are packaged, the function of Cas9 will be more transient than delivered by *Cas9* mRNA, which explains the high on-target/off-target ratio observed with RNP LVLPs; (iii) the RNP LVLPs showed faster action after transduction, and this feature will be especially useful in applications such as generating transgenic animal models, where fast action will decrease the degree of chimerism and increase the chance of germline transmission.

Not only useful for gene disruption, the RNP LVLPs will also be useful for homology-mediated repair, provided that a donor template be provided by other vehicles, such as IDLV or AAV. Although our current system packaged SaCas9 RNP, we believe that similar strategy should be translatable to other editor proteins (e.g. spCas9 and Cas12a) for gene disruption. In addition, it should also be used to package dCas9/sgRNA or nickase/sgRNA for CRISPR interference (CRISPRi), CRISPR activation (CRISPRa) and base editing.

In summary, the present work turns the widely used lentiviral vector into an efficient Cas9 RNP delivery vehicle for transient function and efficient gene editing. In addition, similar strategy may be used for packaging and delivering other RNPs into mammalian cells, which has been difficult to achieve so far.

## DATA AVAILABILITY

All data that support the findings of this study are available in the paper and its Supplementary Information.

## Supplementary Material

gkz605_Supplemental_File
