# Supplementary material for: Delivering Cas9/sgRNA ribonucleoprotein (RNP) by lentiviral capsid-based bionanoparticles for efficient ‘hit-and-run’ genome editing
Source: Nucleic Acids Res. 2019 Jul 12;47(17):e99. doi: 10.1093/nar/gkz605 (PMC6753487; doi:10.1093/nar/gkz605)
Supplement: gkz605_Supplemental_File [file gkz605_supplemental_file.pdf]

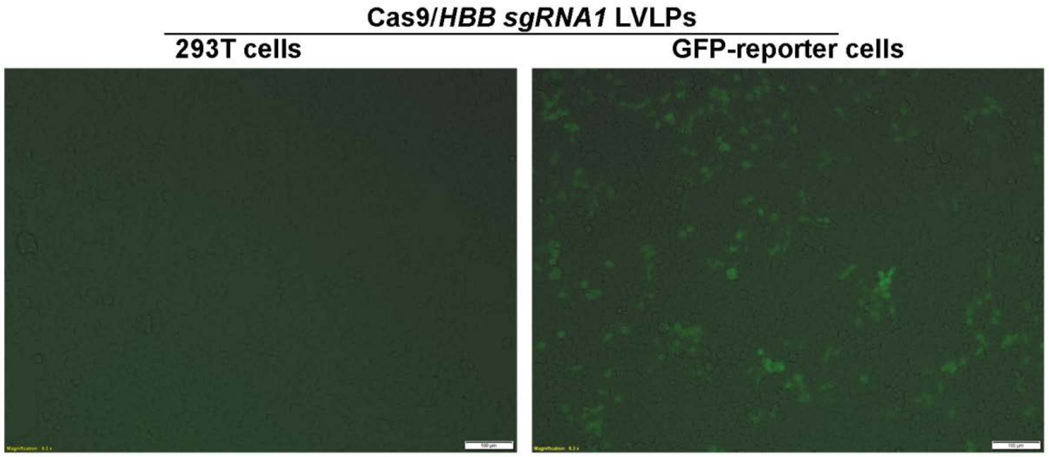

Supplementary Figure S1. The LVLPs generated GFP-positive cells in GFP-reporter cells but not in HEK293T cells. 2.5×10^4 cells were seeded in 24-well plates and 150 ng p24 of Cas9/*HBB sgRNA1* RNP LVLPs were transduced into the cells. The cells were analyzed by fluorescent microscopy 48 hours after transduction.

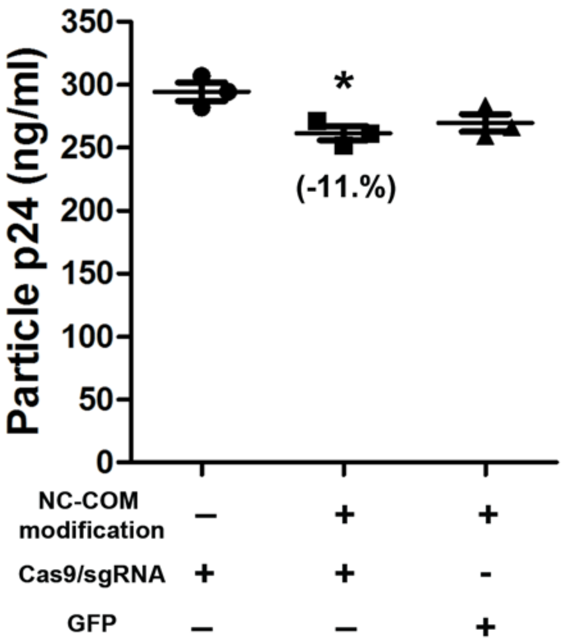

Supplementary Figure S2. Analysis of NC-COM modification on particle assembly efficiency. Packaging plasmid with or without COM modification was used to package Cas9/*HBB sgRNA1*, or GFP lentiviral vector. Transfection was done in 6-well plates; p24 was assayed in supernatants collected between 24-48 hours after transfection. * indicates $p<0.05$ by Tukey's Multiple Comparison Test following ANOVA.

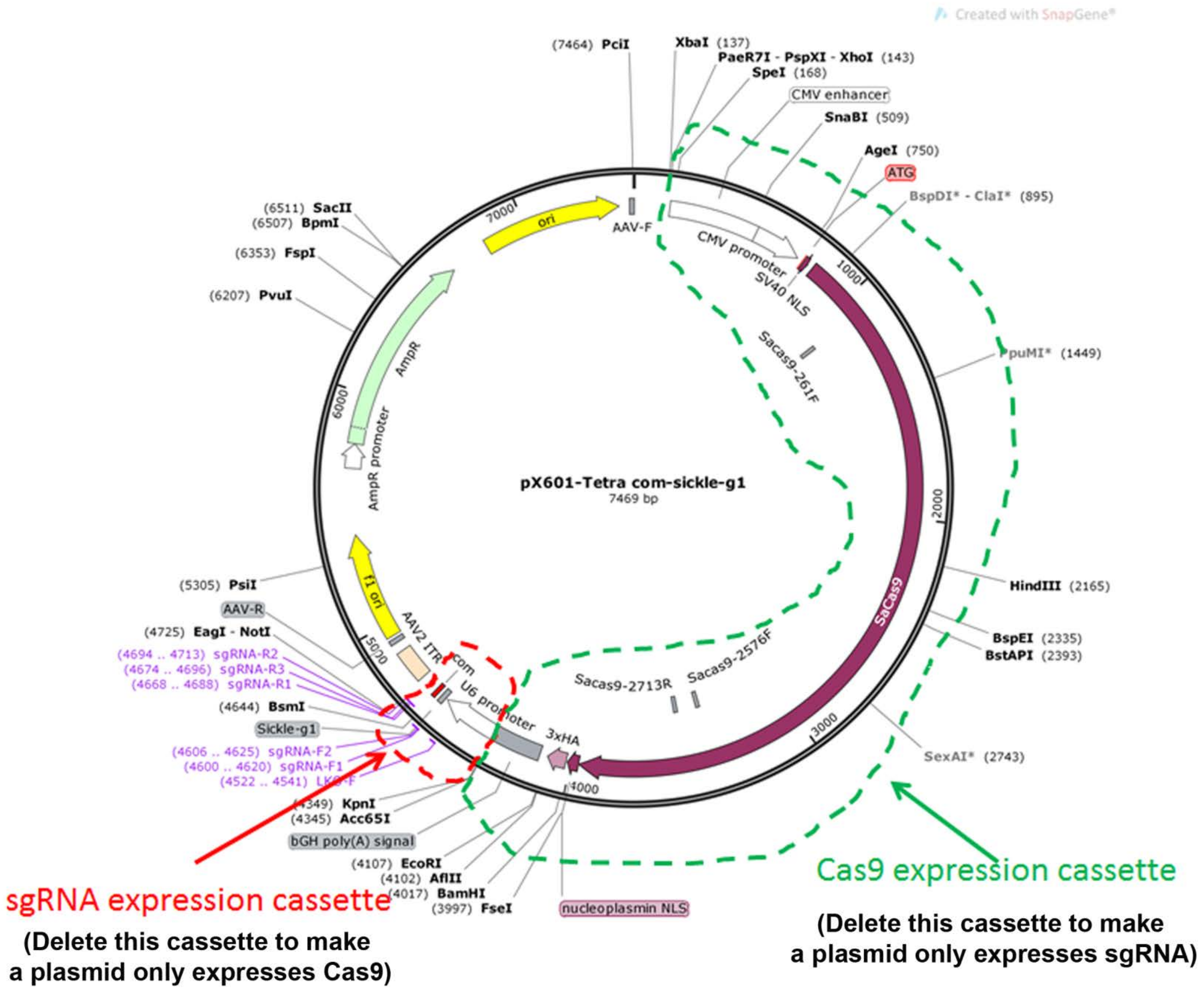

Supplementary Figure S3. Map of the plasmid with both the Cas9- and sgRNA-expression cassettes. Deleting one of the cassettes will make the plasmid only expressing the other product.

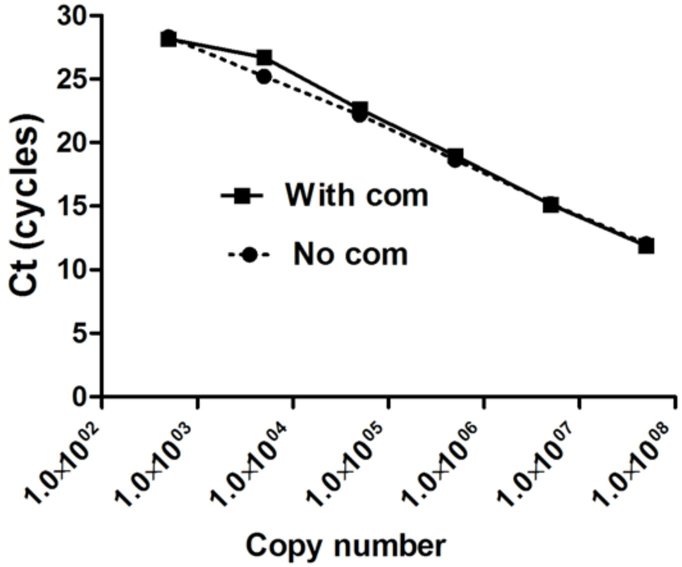

Supplementary Figure S4. Standard curves when the same primer pairs were used to amplify the sgRNA DNA sequence with and without com aptamer.

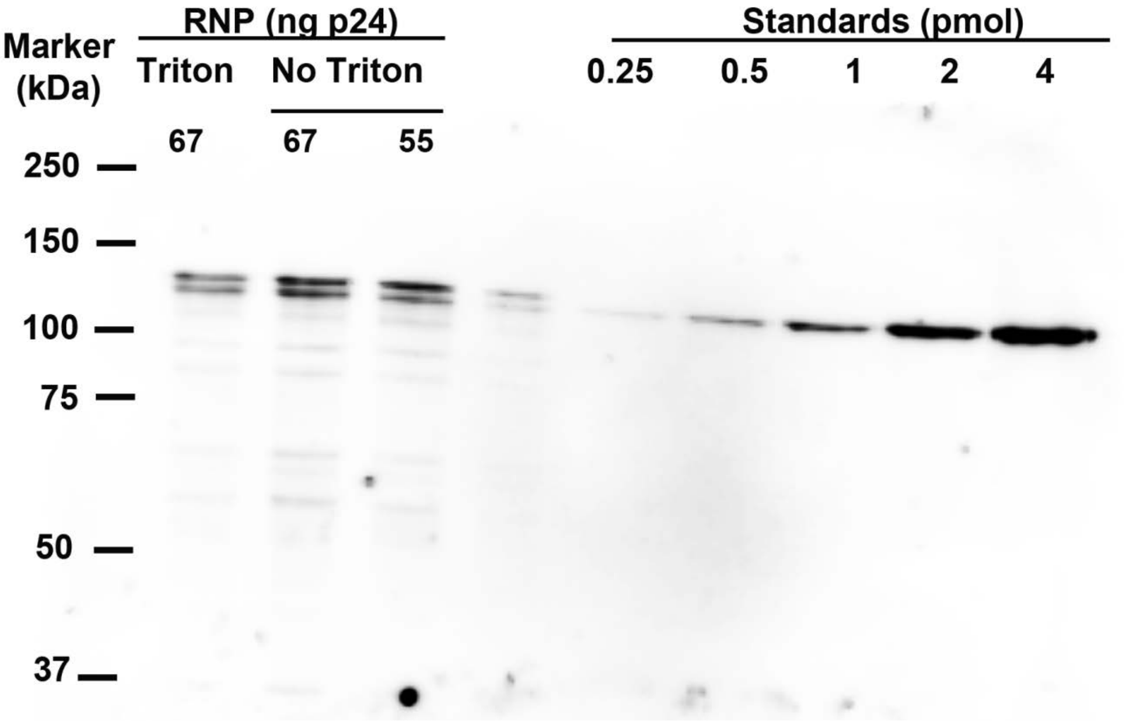

Supplementary Figure S5. Quantification of Cas9 protein in LVLPs. Cas9 RNP LVLPs were treated with or without Triton X-100 and Cas9 protein amount was quantified with purified Cas9 RNP (BioVision Incorporation).

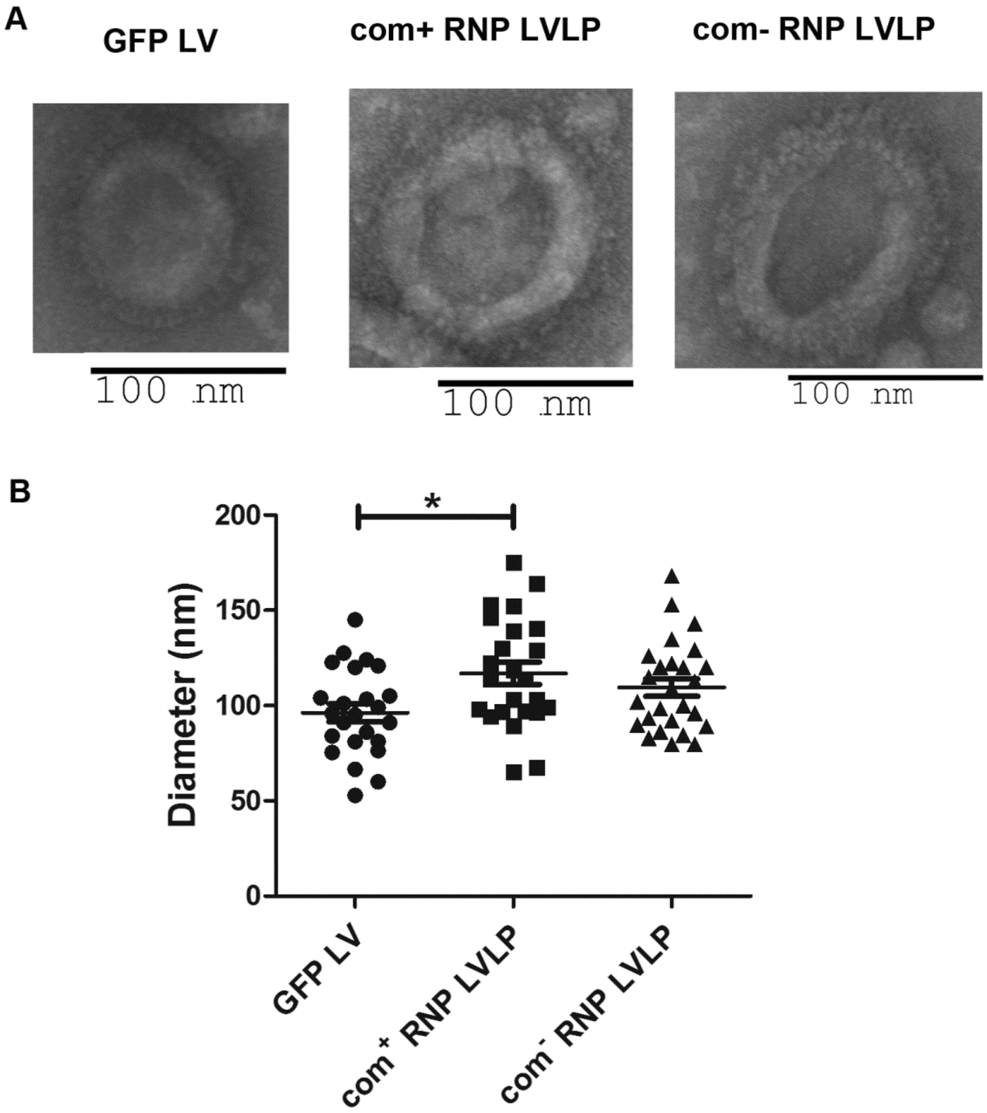

Supplementary Figure S6. Electron microscopy of *com*$^{+}$ and *com*$^{-}$ RNP LVLPs. A. Representative images. GFP LV was GFP-expressing LV packaged with the unmodified packaging plasmid, which has no COM fused to NC. *Com+* RNP LVLPs had *com* modified *HBB* sgRNA1. *Com*$^{-}$ RNP LVLPs had unmodified *HBB* sgRNA1. **B**. Comparison of diameters of different types of particles. Each dot indicates one particle. *, p<0.05 by Tukey's Multiple Comparison Test after one-way ANOVA.

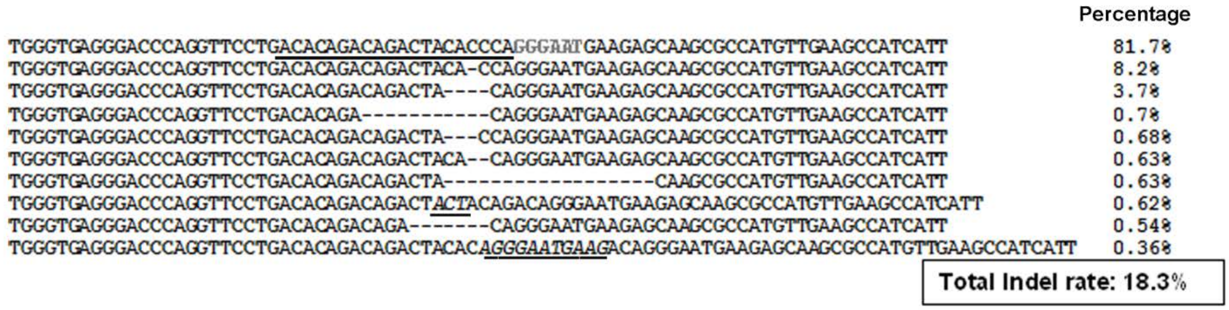

Supplementary Figure S7. Indels generated by Cas9/IL2RG RNP LVLPs in lymphocytes. The nucleotides in gray is the PAM. The nucleotides underlined are the target sequences. The underlined *Italic* nucleotides are insertions.

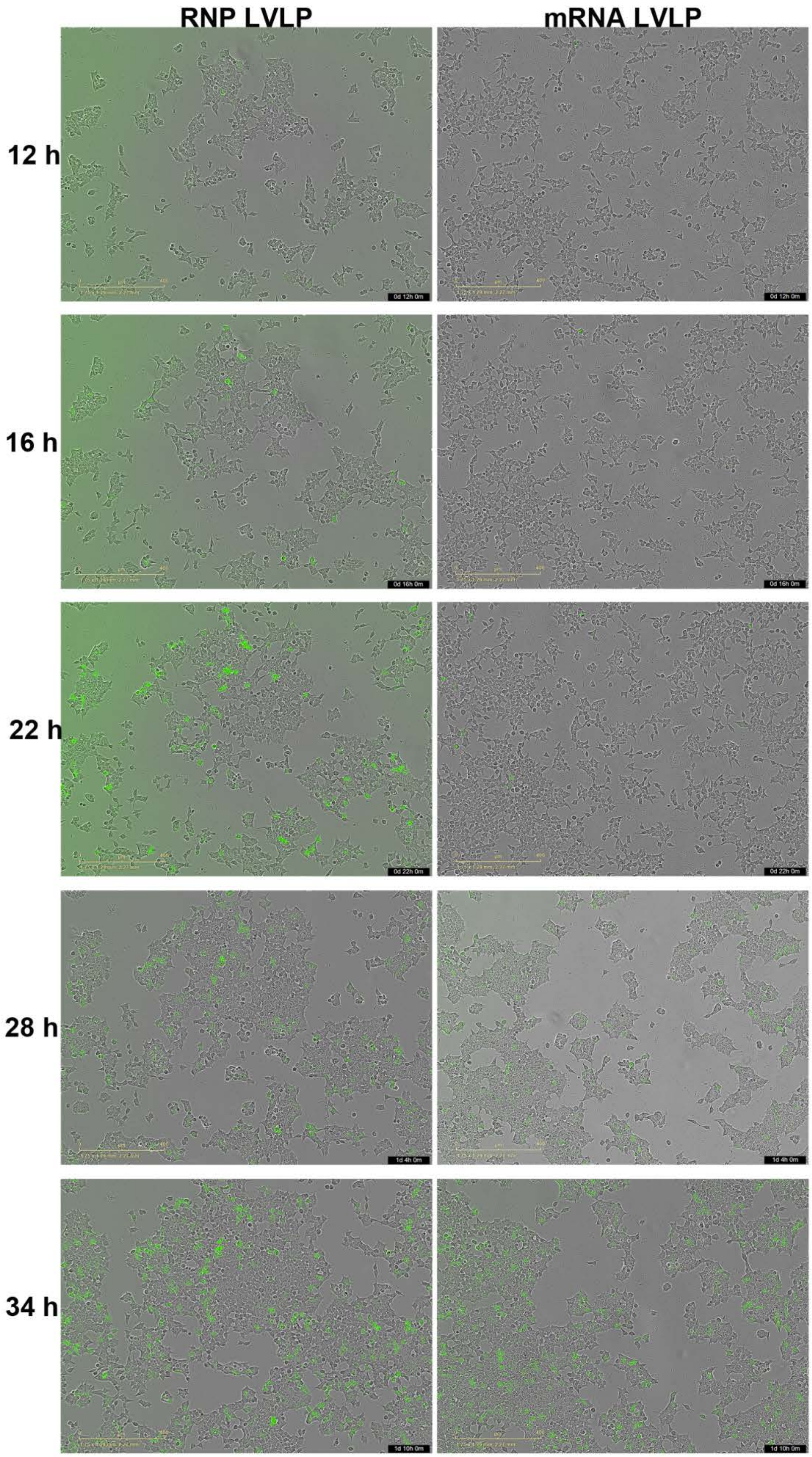

Supplementary Figure S8. Images of kinetics of GFP-expression. Cells transduced with RNP LVLPs showed GFP-positive cells earlier than cells co-transduced with *Cas9* mRNA LVLPs and *HBB* sgRNA1-expressing LVs. At 34 hours after transduction, the two types of cells showed similar rates of GFP-positive cells. Quantitative data of 18 different areas for each cell type were presented in Fig.4E.

Supplementary Table 1. Plasmids Used

No.	Name	Purpose	Generation strategy
**Packaging Plasmids**			
1	psPAX2-D64V-NC-COM-N22	Second generation lentivirus packaging plasmid with COM-lambda N22 peptide inserted after NC ZF2.	The BmgBI and BspEI fragment of psPAX2-D64V-NC-MS2x2 was replaced with a synthesized DNA fragment encoding COM-λ N22 fusion peptide by T4 DNA ligase.<br>DNA sequence:<br>GACGTGatgaaatcaattcgctgtaaaaactgcaacaaactgttatttaag<br>gcggattcctttgatcacattgaaatcaggtgtccgcgttgcaaacgtcacatc<br>ataatgctgaatgcctgcgagcatcccacggagaaacattgtgggaaaagag<br>aaaaaatcacgcattctgacgaaaccgtgcgttatGGCGGTCACGTGTC<br>TTCAGGCGGAGGGATGGGTAATGCTCGGACCCGGCGAAG<br>AGAGAGGCGGGCTGAGAAGCAGGCACAGTGGAAGGCTGC<br>AAACGTTAACTCCGGA<br>(enzyme site-COM-Liner-λ N22-Linker-enzyme site)<br>Amino acids:<br>DVMKSIRCKNCNKLLFKADSFDHIEIRCPRCKRHIIMLNACEHP<br>TEKHCGKREKITHSDETVRYGHVSSGGGMGNARTRRRERRAE<br>KQAQWKAANVNSGGGGG<br>(COM-Liner-λ N22-Linker)
2	psPAX2-D64V-NC-COM	Second generation lentivirus packaging plasmid with one copy of COM inserted after NC ZF2.	The PmlI-HpaI fragment of psPAX2-D64V-NC-COM-N22 was removed by restriction and self-ligation of the backbone, removing λ N22 and keeping COM.<br>DNA sequence:<br>GACGTGatgaaatcaattcgctgtaaaaactgcaacaaactgttatttaag<br>gcggattcctttgatcacattgaaatcaggtgtccgcgttgcaaacgtcacatc<br>ataatgctgaatgcctgcgagcatcccacggagaaacattgtgggaaaagag<br>aaaaaatcacgcattctgacgaaaccgtgcgttatGGCGGTCACAACTC<br>CGGA<br><br>(enzyme site-COM-Liner -enzyme site)<br><br>Amino acids:<br>DVMKSIRCKNCNKLLFKADSFDHIEIRCPRCKRHIIMLNACEHP<br>TEKHCGKREKITHSDETVRYGGHDSGGGGG<br>(COM-Liner)
**sgRNA mammalian Expression Plasmids**			
3	pSaCas9-HBB-sgRNA1^{Tetracom}	Plasmid expressing SaCas9 mRNA and the guide RNA for HBB; a com aptamer was used to replace the tetraloop of the sgRNA.	Acc65I-NotI fragment of pX601-AAV-CMV::NLS-SaCas9-NLS-3xHA-bGHpA;U6::BsaI-sgRNA was replaced by a synthetic DNA fragment (by Genscript Inc) by T4 DNA ligase. This DNA fragment encodes a HBB sgRNA1 with a com aptamer at the Tetraloop position.<br><br>GGTACCGAGGGCCTATTTCCCATGATTCCTTCATATTTGCAT<br>ATACGATACAAGGCTGTTAGAGAGATAATTGGAATTAATTT<br>GACTGTAAACACAAAGATATTAGTACAAAATACGTGACGTA<br>GAAAGTAATAATTTCTTGGGTAGTTTGCAGTTTTAAAATTAT<br>GTTTTAAAATGGACTATCATATGCTTACCGTAACTTGAAAGT<br>ATTTCGATTTCTTGGCTTTATATATCTTGTGGAAAGGACGAA<br>ACACCGAGTAACGGCAGACTTCTCCACGTTTAAGTACTCTG<br>CTGAATGCCTGCGAGCATCCCACCAGAATCTACTTAAACAA<br>GGCAAAATGCCGTGTTTATCTCGTCAACTTGTTGGCGAGAT<br>TTTTTTGCGGCCGC

No.	Name	Purpose	Generation strategy
			(restriction enzyme-U6 promoter-HBB gRNA1-com-restriction enzyme)
4	pSaCas9-HBB-sgRNA1$^{Tetra-PP7}$	Plasmid expressing SaCas9 mRNA and the guide RNA1 for HBB; a PP7 aptamer was used to replace the tetraloop of the sgRNA.	Acc65I-NotI fragment of pX601-AAV-CMV::NLS-SaCas9-NLS-3xHA-bGHpA;U6::BsaI-sgRNA was replaced by a synthetic DNA fragment (by Genscript Inc) by T4 DNA ligase. This DNA fragment encodes a HBB sgRNA1 with a PP7 aptamer replacing the Tetraloop.<br><br>GGTACCGAGGGCCTATTTCCCATGATTCCTTCATATTTGCAT<br>ATACGATACAAGGCTGTTAGAGAGATAATTGGAATTAATTT<br>GACTGTAAACACAAAGATATTAGTACAAAATACGTGACGTA<br>GAAAGTAATAATTTCTTGGGTAGTTTGCAGTTTTAAAATTAT<br>GTTTTAAAATGGACTATCATATGCTTACCGTAACTTGAAAGT<br>ATTTCGATTTCTTGGCTTTATATATCTTGTGGAAAGGACGAA<br>ACACCGAGTAACGGCAGACTTCTCCACGTTTAAGTACTCTG<br>GGAGCAGACGATATGGCGTCGCTCCCAGAATCTACTTAAAC<br>AAGGCAAAATGCCGTGTTTATCTCGTCAACTTGTTGGCGAG<br>ATTTTTTTGCGGCCGC<br><br>(restriction enzyme-U6 promoter-HBB gRNA1-PP7-restriction enzyme)
5	pSaCas9-HBB-sgRNA1$^{Tetra-BoxB}$	Plasmid expressing SaCas9 mRNA and the guide RNA1 for HBB; a BoxB aptamer was used to replace the tetraloop of the sgRNA.	Acc65I-NotI fragment of pX601-AAV-CMV::NLS-SaCas9-NLS-3xHA-bGHpA;U6::BsaI-sgRNA was replaced by a synthetic DNA fragment (by Genscript Inc) by T4 DNA ligase. This DNA fragment encodes a HBB sgRNA1 with a BoxB aptamer replacing the Tetraloop.<br><br>GGTACCGAGGGCCTATTTCCCATGATTCCTTCATATTTGCAT<br>ATACGATACAAGGCTGTTAGAGAGATAATTGGAATTAATTT<br>GACTGTAAACACAAAGATATTAGTACAAAATACGTGACGTA<br>GAAAGTAATAATTTCTTGGGTAGTTTGCAGTTTTAAAATTAT<br>GTTTTAAAATGGACTATCATATGCTTACCGTAACTTGAAAGT<br>ATTTCGATTTCTTGGCTTTATATATCTTGTGGAAAGGACGAA<br>ACACCGAGTAACGGCAGACTTCTCCACGTTTAAGTACTCTG<br>GGGCCCTGAAGAAGGGCCCCAGAATCTACTTAAACAAGGC<br>AAAATGCCGTGTTTATCTCGTCAACTTGTTGGCGAGATTTTT<br>TTGCGGCCGC<br>(restriction enzyme-U6 promoter-HBB gRNA1-BoxB-restriction enzyme)
6	pSaCas9-sgRNA-Tetra-com vector	Vector plasmid expressing SaCas9 mRNA and a guide RNA scaffold with the com aptamer replacing the Tetraloop. Gene-specific guide sequence can be inserted into the BsaI site to make a gene-specific sgRNA expression plasmid.	KpnI-NotI fragment of pX601-AAV-CMV::NLS-SaCas9-NLS-3xHA-bGHpA;U6::BsaI-sgRNA was replaced by a synthetic DNA fragment (by Genscript Inc) by T4 DNA ligase. This DNA fragment contains a U6 promoter, BsaI restriction sites for cloning of the gRNA, sgRNA scaffold with the com aptamer replacing the Tetraloop.<br><br>Ggtaccgagggcctatttcccatgattccttcatatttgcatatacgatacaag<br>gctgttagagagataattggaattaatttgactgtaaacacaaagatattagt<br>acaaaatacgtgacgtagaaagtaataatttcttgggtagtttgcagttttaaa<br>attatgttttaaaatggactatcatatgcttaccgtaacttgaaagtatttcgatt<br>tcttggctttatatatcttgtggaaaggacgaaacaccGGAGACCACGGC<br>AGGTCTCAgtttaagtactctgCTGAATGCCTGCGAGCATCCCAC<br>cagaatctacttaaacaaggcaaaatgccgtgtttatctcgtcaacttgttggc<br>gagatttttttgcggccgc

No.	Name	Purpose	Generation strategy
			(restriction enzyme-U6 promoter-Gene-specific gRNA-com-restriction enzyme)
7	pHBB-sgRNA1$^{Tetra com}$	Plasmid expressing the guide RNA for HBB sgRNA1, with the com aptamer replacing the Tetraloop.	pSaCas9-HBB-sgRNA1$^{Tetra com}$ was cut with XbaI and Acc65I to remove the Cas9 expression cassette. The vector part was blunted by Klenow enzyme and re-ligated.
8	pSaCas9-IL2RG-sgRNA1$^{Tetra com}$	Plasmid expressing SaCas9 mRNA and the guide RNA1 for IL2RG; a com aptamer was used to replace the tetraloop of the sgRNA.	pSaCas9-sgRNA-Tetra-com vector was cut with BasI, and the annealed product between Scid-g2F (CACCGACACAGACAGACTACACCCA) and Scid-g2R (AAACTGGGTGTAGTCTGTCTGTGTC) was inserted between the two BsaI sites by T4 DNA ligase.
9	pIL2RG-sgRNA1$^{Tetra com}$	Plasmid expressing sgRNA IL2RG RNA1; a com aptamer was used to replace the tetraloop of the sgRNA.	pSaCas9-IL2RG-sgRNA1$^{Tetra com}$ was cut with XbaI and Acc65I to remove the Cas9 expression cassette. The vector part was blunted by Klenow enzyme and re-ligated.
10	pCSII-IL2RG-Rep	Lentiviral vector containing two IL2RG sgRNA expression cassettes and a donor template for inserting IL2RG cDNA through homologous recombination.	Replace the AgeI and NotI fragment of pCSII-EF-miRFP709-hCdt(1/100) (addgene Plasmid #80007) with a synthesized AgeI-NotI fragment (Genscript Inc), which contains the *IL2RG* 5' homologous arm, codon optimized *IL2RG* cDNA, *IL2RG* 3' homologous arm, and two U6 promoter-driven *IL2RG* sgRNA expression cassettes.<br><br>gcggccgcTCTCGAACTCCTCAAGCAATCCACCTGC<br>CTTGGCCTCCCAAAGTGCTGGGATTGCAGGCGT<br>GAGTCACTGCACCCAGCCGAGAGAATAAATTTC<br>TGTTGGTTTAAGCCACTCAGTTTGGGGATAACTT<br>ATGGCAGCCCTAGCAAACTAATACATACTAAAG<br>ATACATACTAAATACTAAGCTGGGCCATATAGT<br>CCAGTTTTCCTGAGACTCCCAGGCAAGTGCTGTT<br>TTTCTTTGCTTAATATCCTACACCACTTTCTGTCT<br>GGTAAAATTACACTCATTCTTTAAGATGCCACTG<br>AAATAGCACCTCTTCAGCACAGCCTTCACTAAA<br>CTATCCCCCTCTCCATCTTGGTAAATTTAGTTAC<br>TTCCTCTTCTGTGCTCACATACTTTGTAGTATCTC<br>TACATTTATGCTATAGGACTTGTTACACTATGTT<br>GTATTACTTGTTTATGTCTTCCCCACTTTTCTGTG<br>AGTGTCTAGAAATATGAGGATGTCTTGTTGGTCT<br>ATTTCCAGAACATAAGCACAGTGCCTGGCACAT<br>ATTAAAAACGTAATAAATGTTTGCTGAATAAAT<br>AGTTTCTGTAAGTGGCTTCTCCAATCACCTCTGT<br>GTTTTCGGGGAAGGTAAAACTGGCAACAGGATG<br>AAGAATGGATTAGAGAGCAGAGGGCCTTTAGAA<br>AGGGAGGCCAGTTGATGGAGTCTAGATAGAATC<br>ATGACTAGAGCTAATGAAAGACTGATTTAGCAG<br>AGTGGCTGTGGTAATGGAAAGGAGGAAACCGTT<br>GGGAGAAACACCACAGAAGCAGAGTGGGTTAT<br>ATTCTCTGGGTGAGAGAGGGGGAGAAATTGAAG<br>CTGATTCTGAGGTTTCAAGTCTGGGTGACTGAGA<br>GGGTGACGATACCATTGACTGAGGTGGGGAAGG<br>CAGGAAGAGAAGCAGAGTTGGGGGAAGATGGG<br>AAGCTTGAAGCTAGTATTGTTGTTCCTCCATTTC<br>TAGAATATTTTTGTATTATAAGTCACACTTCCTC<br>GCCAGTCTCAACAGGGACCCAGCTCAGGCAGCA

No.	Name	Purpose	Generation strategy
			GCTAAGGGTGGGTATTCTGGTTTGGATTAGATCA<br>GAGGAAAGACAGCTGTATATGTGCCCACAGGAG<br>CCAAGACGGTATTTTCCATCCTCCCAAAACAGTA<br>GAGCTTTGACAGAGATTTAAGGGTGACCAAGTC<br>AAGGAAGAGGCATGGCATAGAACGGTGATGTCG<br>GGGGTGGGGGTTCAGAACTTCCATTATAGAAGG<br>TAATGATTTAGAGGAGAAGGTGGTTGAGAATGG<br>TGCTAGTGGTAGTGAACAGATCCTTCCCAGGAT<br>CTAGGTGGGCTGAGGATTTTTGAGTCTGTGACAC<br>TATTGTATATCCAGCTTTAGTTTCTGTTTACCACC<br>TTACAGCAGCACCTAATCTCCTAGAGGACTTAG<br>CCCGTGTCACACAGCACATATTTGCCACACCCTC<br>TGTAAAGCCCTGGTTTATAAGGTTCTTTCCACCG<br>GAAGCTATGACAGAGGAAACGTGTGGGTGGGG<br>AGGGGTAGTGGGTGAGGGACCCAGGTTCCTGccA<br>ccatgCtCaaAccTtcCCtGccTttTacCAGcTtGCtGttTctCca<br>gctCccTctCctCggCgtCggActCaaTacAacTatCctGacAccT<br>aaCggAaaCgaGgaTacAacCgcCgaCttTttTctCacAacCatgc<br>cTacAgaTAGcTtGTCCgtGAGcacCctCccTctGccTgaAgt<br>GcagtgCttCgtCttTaaCgtGgaAtaTatgaaCtgTacCtggaaTT<br>CcTCcAGCgaAccTcagccAacAaaTctGacActCcaCtaCtgg<br>taTaaAaaTAGCgaCaaCgaCaaGgtGcagaaAtgTTCccaTta<br>CTTGttTAGCgaGgaGatTacCAGCggAtgCcagCtCcaGaa<br>GaaAgaAatTcaTctGtaTcaGacCttCgtGgtGcagctGcaggaT<br>ccTAgAgaGccTagAagGcaggcTacCcagatgTTGaaGctCca<br>gaaCctCgtCatTccTtgggcCccTgaAaaTctGacCTtGcaTaaG<br>ctCTCCgaGAGccagctGgaGctCaaTtggaaTaacagGttTCtg<br>aaTcaTtgCCtggaAcaTCTCgtCcaAtaTAGAacCgaTtggga<br>TcaTTCctggacCgaGcaGAGCgtCgaCtaCCgGcaCaaAttT<br>AGcCtgccAagCgtCgaCggAcagaaGAgAtaTacCttCAgAg<br>tGAgATCcAgAttCaaTccTctGtgCggCTCCgcAcagcaCtg<br>gTCCgaGtggTCccaTccTatTcaTtggggATCcaaCacCAGC<br>aaGgaAaaCccAttTctgttCgcTCTggaGgcTgtCgtGatTAGC<br>gtGggAAGcatgggCCtgatCatTTCcTTGctGtgCgtTtaCttTt<br>ggctggaGAgAacCatgccTAgGatCccTacActCaaAaaTctGg<br>aAgaCTTGgtGacAgaGtaTcaTggAaaTttCAGCgcTtggTC<br>CggAgtCAGCaaAggCctCgcCgaAagCctCcagccTgaTtaT<br>agCgaGAgGctGtgTctGgtGagCgaAatCccTccTaaGggCgg<br>AgcTctGggCgaAggAccAggCgCTAGCCCTTGTAATC<br>AGCACTCCCCTTATTGGGCTCCTCCTTGCTATAC<br>ATTGAAACCAGAGACATAAGGGAACCCAGGAG<br>ACAGGCCACACAGATGCTAAAACTGCAGAATCT<br>GGGTAATTTGGAAAGAAAGGGTCAAGAGACCA<br>GGGATACTGTGGGACATTGGAGTCTACAGAGTA<br>GTGTTCTTTTATCATAAGGGTACATGGGCAGAA<br>AAGAGGAGGTAGGGGATCATGATGGGAAGGGA<br>GGAGGTATTAGGGGCACTACCTTCAGGATCCTG<br>ACTTGTCTAGGCCAGGGGAATGACCACATATGC<br>ACACATATCTCCAGTGATCCCCTGGGCTCCAGA<br>GAACCTAACACTTCACAAACTGAGTGAATCCCA<br>GCTAGAACTGAACTGGAACAACAGATTCTTGAA<br>CCACTGTTTGGAGCACTTGGTGCAGTACCGGACT<br>GACTGGGACCACAGCTGGACTGTGAGTGACTAG<br>GGACGTGAATGTAGCAGCTAAGGCCAAGAAAGT<br>AGGGCTAAAGGATTCAACCAGACAGATAGAAG<br>GACCTAATATCAAGCTCCTGTTCTCTGCCTCCCA<br>GCTTCTCTGCTCACCCCCTACCCTCCCTCCTCCA<br>ACTCCTTTCCTCGAGGGCCTATTTCCCATGATTC<br>CTTCATATTTGCATATACGATACAAGGCTGTTAG

No.	Name	Purpose	Generation strategy
			AGAGATAATTGGAATTAATTTGACTGTAAACAC AAAGATATTAGTACAAAATACGTGACGTAGAAA GTAATAATTTCTTGGGTAGTTTGCAGTTTTAAAA TTATGTTTTAAAATGGACTATCATATGCTTACCG TAACTTGAAAGTATTTCGATTTCTTGGCTTTATA TATCTTGTGGAAAGGACGAAACACCGTGGCCTG TCTCCTGGGTTCCCGTTTTAGTACTCTGGAAACA GAATCTACTAAAACAAGGCAAAATGCCGTGTTT ATCTCGTCAACTTGTTGGCGAGATTTTTTGTTTT AGAGCTAGAAATAGCAAGTTAAAATAAGGCTAG TCCGTTTTTAGCGCGTGCGCCAATTCTGCAGACA AATGAGGGCCTATTTCCCATGATTCCTTCATATT TGCATATACGATACAAGGCTGTTAGAGAGATAA TTGGAATTAATTTGACTGTAAACACAAAGATATT AGTACAAAATACGTGACGTAGAAAGTAATAATT TCTTGGGTAGTTTGCAGTTTTAAAATTATGTTTT AAAATGGACTATCATATGCTTACCGTAACTTGA AAGTATTTCGATTTCTTGGCTTTATATATCTTGTG GAAAGGACGAAACACCGGACACAGACAGACTA CACCCAGTTTTAGTACTCTGGAAACAGAATCTAC TAAAACAAGGCAAAATGCCGTGTTTATCTCGTC AACTTGTTGGCGAGATTTTTggtAccggt<br><br>cDNA encoded IL2RG amino acid sequence:<br>MLKPSLPFTSLLFLQLPLLGVGLNTTILTPNGNEDT TADFFLTTMPTDSLSVSTLPLPEVQCFVFNVEYMN CTWNSSSEPQPTNLTLHYWYKNSDNDKVQKCSHY LFSEEITSGCQLQKKEIHLYQTFVVQLQDPREPRRQ ATQMLKLQNLVIPWAPENLTLHKLSESQLELNWN NRFLNHCLEHLVQYRTDWDHSWTEQSVDYRHKF SLPSVDGQKRYTFRVRSRFNPLCGSAQHWSEWSH PIHWGSNTSKENPFLFALEAVVISVGSMGLIISLLC VYFWLERTMPRIPTLKNLEDLVTEYHGNFSAWSG VSKGLAESLQPDYSERLCLVSEIPPKGGALGEGPG ASPCNQHSPYWAPPCYTLKPET<br><br>Color Key:<br>Restriction enzyme-5’ arm-IL2RG cDNA-3’ arm-U6-IL2RG sgRNA1-U6-IL2RG sgRNA2- Restriction enzyme.

Supplementary Table 2. Primers Used for the study

Primer name	SEQ	Use
sgRNA-F1	GAGTAACGGCAGACTTCTCCA	For PCR detection of *HBB sgRNA1* with sgRNA-R1.
sgRNA-R1	CGGCATTTTGCCTTGTTTAAG	For PCR detection of *HBB sgRNA1* with sgRNA-F1.
sgRNA-R2	CGCCAACAAGTTGACGAGAT	Used as a gene-specific primer in reverse transcription to detect sgRNA
sgRNA-R3	GATAAACACGGCATTTTGCCTTG	For PCR detection of *HBB sgRNA1* with sgRNA-F1.
Reporter-F	tccatttcaggtgtcgtgag	To amplify the DNA in the GFP reporter cassette with Reporter-R1.
Reporter-R1	GAACTTCAGGGTCAGCTTGC	To amplify the DNA in the GFP reporter cassette with Reporter-F.
IL2RG-1029F	ATGCCCTCTGTAGTGGGTTG	Used with IL2RG-3301R to amplify the 5′ IL2RG region. The primers could not amplify from the donor template.
IL2RG-3301R	GGCAGCTGCAGGAATAAGAG	Used with IL2RG-1029F to amplify the 5′ IL2RG region. The primers could not amplify from the donor template.
IL2RG-R4-H	gGagctgGagAaaCaGCaAg	Used with IL2RG-F to amplify 5′ junction after cDNA insertion by homologous recombination for NGS.
IL2RG-F	CACAGCACATATTTGCCACACCC	Used with IL2RG-R4-H to amplify 5′ junction after cDNA insertion by homologous recombination for NGS. It was also used with IL2RG-3301R to amplify the IL2RG DNA without homologous recombination for NGS.
IL2RG-4380F	CACTTCTGGCTGTCAGTTGC	Used with IL2RG-5354R to amplify the 3′ IL2RG gDNA. The primers could not amplify from the donor template.
IL2RG-5354R	CACTGGTGTCAGGAATGGTG	Used with IL2RG-4380F to amplify the 3′ IL2RG gDNA. The primers could not amplify from the donor template.
IL2RG-4580R	TCTTTTCTGCCCATGTACCC	Used with IL2RG-4380F to amplify the unmodified IL2RG 3′ DNA.
IL2RG-VF	gcTccTccTtgCtaTacATTG	Used with IL2RG-4580R to amplify the IL2RG 3′ junction DNA after cDNA insertion by homologous recombination.
HBB-1849F	CGATCACGTTGGGAAGCTATAGAG	Used with HBB-5277R to amplify the HBB region from human cells. The primers could not amplify from the donor template.
HBB-5277R	AACATCCTGAGGAAGAATGGGAC	Used with HBB-1849F to amplify the HBB region from human cells. The primers could not amplify from the donor template.
HBB-R2	CCAATAGGCAGAGAGAGTCAGTG	Used with HBB-F2 to amplify the HBB region for NGS.
HBB-F2	GTTCACTAGCAACCTCAAACAG	Used with HBB-R2 to amplify the HBB region for NGS.
HHB-R	GGCATAAAAGTCAGGGCAGAGC	Used to amplify the HBB region for NGS with HBB-R3
HBB-R1	AGCCAGGGCTGGGCATAAAAG	Used to amplify the HBB region for NGS with HBB-R3
HBB-R3	TGGGAAAATAGACCAATAGGCAGAG	Used to amplify the HBB region for NGS with HBB-R or HBB-R

Supplementary Table 3. Comparison of on target and off-target Indel rate of Cas9/*HBB sgRNA1* RNP LVLPs with those of other delivery vehicles[a].

	On-target Indel rate (%)	Off-target Indel rate (%)	On/off target ratio (On-target Indel/Off-target Indel)
**RNP LVLP**	35.5	0.9	39.4
**mRNA LVLP**	86.5	8.0	10.8
**LV**	88.9	71.2	1.25
**IDLV**	60.3	14.2	4.25
**AAV**	50.1	5.4	9.28
**DNA transfection**	66.9	21.4	3.1

[a]The GFP-reporter transgene region (contains the on-target) and endogenous HBB gene region (the off-target with 1 nt mismatch) were amplified and sequenced by NGS to obtain the On-target and the Off-target Indel rates for each sample. The on-target to off-target Indel rate ratios ("on/off" ratios) are the results of on-target Indel rates divided by off-target Indel rates. Cells were harvested 72 hours after treatment. For cells grown in 24-well plates, 1.25×10^5 and 2.5×10^4 cells were used for transfection and transduction respectively. For RNP LVLPs, 55 ng p24 were used (these particles were un-concentrated with 110 ng p24/ml, 55 ng was the maximal amount that we could add due to the relative low concentration); for mRNA LVLPs, 45 ng p24 of *Cas9* mRNA LVLPs and 60 ng p24 of *HBB sgRNA1*-expressing IDLV were used; for LV co-expressing Cas9 and *HBB sgRNA1*, 30 ng p24 were used; for IDLV co-expressing Cas9 and *HBB sgRNA1*, 30 ng p24 were used; for Cas9-expressing AAV6 and *HBB sgRNA1*-expressing AAV6, 10^4 virus genome/cell were used for each virus.
